# Diets high in corn oil or extra-virgin olive oil differentially modify the gene expression profile of the mammary gland and influence experimental breast cancer susceptibility

**DOI:** 10.1007/s00394-015-0958-2

**Published:** 2015-06-20

**Authors:** Raquel Moral, Raquel Escrich, Montserrat Solanas, Elena Vela, M. Carme Ruiz de Villa, Eduard Escrich

**Affiliations:** Physiology Unit, Department of Cell Biology, Physiology and Immunology, Faculty of Medicine, Universitat Autònoma de Barcelona, Bellaterra, 08193 Barcelona, Spain; Department of Statistics, Universitat de Barcelona, 08028 Barcelona, Spain

**Keywords:** Extra-virgin olive oil, Corn oil, Dietary lipids, Mammary gland, Experimental tumors, Gene expression profile

## Abstract

**Purpose:**

Nutritional factors, especially dietary lipids, may have a role in the etiology of breast cancer. We aimed to analyze the effects of high-fat diets on the susceptibility of the mammary gland to experimental malignant transformation.

**Methods:**

Female Sprague–Dawley rats were fed a low-fat, high-corn-oil, or high-extra-virgin olive oil (EVOO) diet from weaning or from induction. Animals were induced with 7,12-dimethylbenz[a]anthracene at 53 days and euthanized at 36, 51, 100 and 246 days. Gene expression profiles of mammary glands were determined by microarrays. Further molecular analyses were performed by real-time PCR, TUNEL and immunohistochemistry. Carcinogenesis parameters were determined at 105 and 246 days.

**Results:**

High-corn-oil diet increased body weight and mass when administered from weaning. The EVOO diet did not modify these parameters and increased the hepatic expression of UCP2, suggesting a decrease in intake/expenditure balance. Both diets differentially modified the gene expression profile of the mammary gland, especially after short dietary intervention. Corn oil down-regulated the expression of genes related to immune system and apoptosis, whereas EVOO modified the expression of metabolism genes. Further analysis suggested an increase in proliferation and lower apoptosis in the mammary glands by effect of the high-corn-oil diet, which may be one of the mechanisms of its clear stimulating effect on carcinogenesis.

**Conclusions:**

The high-corn-oil diet strongly stimulates mammary tumorigenesis in association with modifications in the expression profile and an increased proliferation/apoptosis balance of the mammary gland.

**Electronic supplementary material:**

The online version of this article (doi:10.1007/s00394-015-0958-2) contains supplementary material, which is available to authorized users.

## Introduction

Breast cancer is a leading cause of mortality in women worldwide [1]. Although the etiology of this neoplasia is clearly multifactorial, there is striking evidence of the influence of environmental factors, diet being one of the most important [2]. Human and especially experimental data have shown the effects of dietary lipids on breast cancer risk [2–4]. Epidemiological studies have reported some inconsistent data, likely because of the difficulties in precisely estimating fat intake [5]. However, new approaches from the European Prospective Investigation into Cancer and Nutrition (EPIC) have found significant positive associations of saturated and total fat intake with breast cancer risk [6, 7]. On the other hand, animal models have provided a wealth of evidence for the relationship between dietary lipids and breast cancer. Thus, n-6 polyunsaturated fatty acids (PUFA), particularly linoleic acid (18:2n-6), have shown a strong stimulating effect on breast, colorectal and prostate cancers. Saturated fat, mainly from animal origin, has also a stimulating influence on tumorigenesis. On the contrary, n-3 PUFA, conjugated linoleic acid and γ-linolenic acid, have demonstrated an inhibitory influence on carcinogenesis. The effects of monounsaturated fatty acids (MUFA) are still not well elucidated, although several studies reported a potential protective effect on experimental breast cancer [3, 4]. In this sense, there is also epidemiological evidence that Mediterranean diet (characterized by the consumption of olive oil as the principal source of fat) has a protective effect on the risk of developing several types of tumors, including breast cancer [8, 9]. Lifestyle patterns including diet, physical activity and body weight have been associated with metabolic dysregulation, which may play a role in the association between lifestyle and the development of breast cancer. Actually, Mediterranean diet has also been inversely associated with body weight, adiposity and metabolic syndrome, in addition to other chronic diseases [10, 11].

The mammary gland, unlike other organs, is not fully developed after birth. Through postnatal life, the female breast tissue undergoes several key developmental stages, being one of the most important puberty. There is extensive evidence that nutritional status and body size may contribute to maturation trends and breast cancer. Western countries lifestyle, characterized by high consumption of fat, has been associated with obesity and earlier pubertal maturation, known factors of breast cancer risk [2, 12]. The effects of environmental factors, including nutritional ones, are dependent on the developmental stage when exposition occurs. In this regard, the influence of high-fat diets on experimental breast cancer risk may be different if dietetic intervention occurs in utero, before puberty or after sexual maturation [13, 14]. Thus, high-fat diets may affect mammary development and thus the window where the gland is most susceptible to malignant transformation. Furthermore, in experimental models, dietary lipids may differentially influence the initiation (if exposure begins before carcinogen induction) or the promotion (if exposure begins after induction) of tumorigenesis [3].

The different effects that diets have on breast cancer susceptibility are probably driven by multiple and complex molecular mechanisms. Although such mechanisms are not fully understood, several have been suggested, including alterations of hormonal status, modifications of the structure and function of membranes, disruption of cell-signaling pathways and modulation of gene expression [4]. It is well known that dietary long-chain fatty acids modulate the transcription of genes involved in lipid metabolism. Although fewer data are published regarding the effects of lipids in cancer-related genes, there is a growing body of evidence about their role in the modulation of genes with a role in proliferation, differentiation and apoptosis [15]. Hence, the aim of this work is to analyze the influence of different high-fat diets, i.e., rich in corn oil or in extra-virgin olive oil (EVOO), administered in different periods (after weaning onwards or after induction onwards) on breast cancer susceptibility and to get insight into the underlying molecular mechanisms. For this extent, developmental changes have been monitored, including growth, body mass indexes and hepatic expression of metabolism genes at different ages. Gene expression profile of breast tissues has been determined at different developmental stages, including peri-puberty and adulthood. Experimental tumors have been induced with DMBA at 53 days of age, and clinical manifestation of carcinogenesis has been analyzed at different ages.

## Materials and methods

### Diets

Three semisynthetic diets were designed: a low-fat diet (at 3.71 kcal/g, containing 7.3 % calories under the form of fat) and two high-fat diets (at 4.56 kcal/g, containing 39.5 % calories in the form of fat). The control low-fat diet contained 3 % corn oil (w/w), the high-corn-oil diet contained 20 % of this same oil, while the high olive oil diet contained 3 % corn oil + 17 % extra-virgin olive oil. Carbohydrates in the form of dextrose were 67.9 %—w/w—(73 % calories) in the low-fat diet and 45.9 %—w/w—(40.3 % calories) in both high-fat diets. They also contained 20 % of calories from proteins (18 g casein/100 g low-fat diet; 23 g casein/100 g high-fat diet) and 5 %—w/w—cellulose, 5.9 %—w/w—salt mixture and 0.24 %—w/w—vitamin mixture (Supplementary Table 1). In order to maintain the normal lipidic metabolism, they were supplemented with methionine (0.51 %—w/w—in the low-fat and 0.66 % in the two high-fat diets), choline (1800 mg/kg diet) and folic acid (5 mg/kg diet). Diets were prepared weekly and stored under nitrogen in the dark at 4 °C. The definition, preparation and suitability of the diets have been previously described [16–19].

### Animals and experimental design

**Fig. 1:**
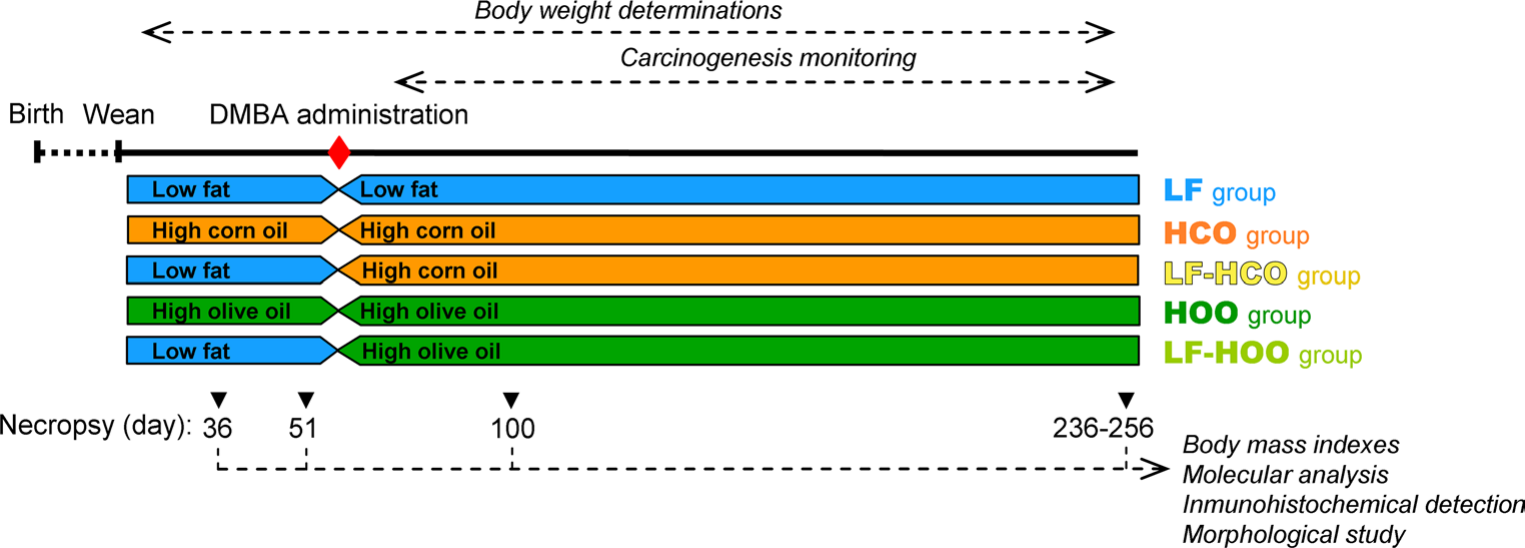
Experimental design. Female Sprague–Dawley rats were fed the low-fat control diet (LF), the high-corn-oil diet from weaning (HCO) or from induction (LF-HCO), and the high-extra-virgin olive oil diet from weaning (HOO) or from induction (LF-HOO). Animals were induced with 5 mg of dimethylbenz[α]anthracene (DMBA) at 53 days of age. Rats were euthanized at day 36 (*N* = 6/experimental condition: LF, HCO, HOO), day 51 (*N* = 6/experimental condition: LF, HCO, HOO), day 100 (*N* = 5/group) and at the end of the assay (236–256, median day 246; *N* = 20/group)

All animals received humane care under an institutionally approved experimental animal protocol, following the legislation applicable in this country. Female Sprague–Dawley Crl:SD rats (*N* = 167; Charles River Lab, L’Arbresle Cedex, France) were distributed depending on the type and timing of dietary intervention. Thus, from weaning onwards, control animals were fed the low-fat diet (group LF, *N* = 87), while the high-fat groups animals were fed the high-corn-oil diet (group HCO, *N* = 37) or the high-extra-virgin olive oil diet (group HOO, *N* = 37) and water ad libitum. At 53 days of age, mammary cancer was induced by oral gavage with one single dose of 5 mg of DMBA (Sigma-Aldrich Inc, St Louis, MO, USA) dissolved in corn oil. To study the promotion of the carcinogenesis, after DMBA treatment, 50 rats from the LF group were changed to high-fat diets (groups LF-HCO, and LF-HOO, *N* = 25 each) (Fig. 1).

Animals were examined and weighed weekly throughout the study. At days of killing, nose-to-anus length was also measured and the following body mass indexes were determined: BMI (body mass index, g/cm^2^) and Lee index $$( {10{,}000 \times {\text{g}}^{{{\raise0.7ex\hbox{$1$} \!\mathord{\left/ {\vphantom {1 3}}\right.\kern-0pt} \!\lower0.7ex\hbox{$3$}}}} /{\text{cm}}} )$$ [20]. From day 74 onwards, all rats were monitored for mammary tumor appearance weekly. Carcinogenesis was evaluated at 105 days (52 days after DMBA induction) and at 236–256 days (median day 246—day 193 post-induction, end of the assay). The following carcinogenesis parameters were calculated: tumor incidence (percentage of tumor-bearing animals), tumor yield (total number of mammary tumors) and total tumor volume (*V* = 4/3π [*d*_1_/2] × [*d*_2_/2]^2^, where* d*_1_ and* d*_2_ are the two diameters of the tumor (*d*_1_ > *d*_2_), and at killing with the three diameters *V* = 4/3π [*d*_1_/2] × [*d*_2_/2] × [*d*_3_/2]).

Rats were euthanized by decapitation at day 36 (*N* = 6/experimental condition: control, HCO, HOO), 51 (*N* = 6/experimental condition: control, HCO, HOO), 100 (*N* = 5/group) and the end of the assay (*N* = 20/group). Left abdominal mammary glands and liver were collected and flash-frozen for molecular analyses. Right mammary glands were fixed in buffered formalin for morphological and histological analyses. At the end of the assay, tumors were excised and a portion fixed in 4 % formalin for histopathological diagnosis [21]. Only data from confirmed mammary adenocarcinomas have been included in this study.

### RNA isolation and gene expression analysis by real-time PCR

Total RNA from liver and left abdominal mammary gland was extracted using the Tissue RNeasy Extraction Kit (QiaGen, Hilden, Germany). RNA was quantified spectrophotometrically with Nanodrop 1000 (ThermoFisher Scientific Inc, Waltham, MA, USA), and integrity was determined using a 2100 Bioanalyser (Agilent Technologies, Santa Clara, CA, USA). The study of expression of specific genes was performed by real-time PCR in the iCycler MyiQ Real-Time PCR detection system (Bio-Rad Laboratories, Hercules, CA, USA). Analyses of mRNA levels were performed using the TaqMan methodology (Applied Biosystems Inc, Foster City, CA, USA). Two micrograms of total RNA was reverse-transcribed using the High Capacity cDNA Reverse Transcription Kit (Applied Biosystems). Reactions were prepared with the TaqMan Universal PCR Master Mix and the suitable TaqMan assay. For the study of metabolism genes, the following assays were used: Rn00566193_m1 (PPARα), Rn00580702_m1 (CPT1a), Rn00664587_m1 (L-FABP), Rn01754856_m1 (UCP2). For validation of arrays (see below), the following assays were used (in alphabetical order of the target gene): Rn00573474_m1 (Acaca), Rn00566411_m1 (Acly), Rn00595250_m1 (Adipoq), Rn01507624_m1 (Cd19), Rn00565469_m1 (Cd28), Rn00565890_m1 (Cd3d), Rn00560963_s1 (Cebpa), Rn01524626_m1 (Csn2—βcasein), Rn01511686_g1 (Hsp90ab1), Rn00574380_m1 (Insig1), Rn00565158_m1 (Lep), Rn00575662_m1 (Pgr), Rn00440945_m1 (PPARγ), Rn00821474_g1 (S100a6) Rn00565937_m1 (Tgfβ3), Rn00710369_m1 (Tp53inp1). Twenty nanograms of cDNA was amplified during 40 cycles of 15 s at 95 °C and 60 s at 60 °C. Gene expression was normalized using Hprt (Rn01527840_m1) as a control transcript.

### Whole-genome gene expression profiling

After quality control of total RNA using 2100 Bioanalyser (Agilent Technologies), only samples with integrity numbers ≥8 were used for these analyses. For the determination of the effects of high-fat diets on the gene expression profile of the mammary gland at different ages, we used GeneChip^®^ Rat Exon 1.0 ST Array (Affymetrix, Santa Clara, CA, USA). Three mammary glands of each group at each age tested were chosen. For each sample, 3 μg of total RNA was labeled, hybridized to chips and scanned, in the Microarrays Service from Vall d’Hebron Research Institute (VHIR).

### Microarray data analysis

The scanned images were processed with Microarray Analysis Suite 5.0 (Affymetrix). Raw expression values were preprocessed using the RMA method [22] for background correction, normalization and summarization of probe values. The data obtained were then non-specific filtered to remove genes with low signal and low variability. The statistical analysis of data was performed using the free statistical language R (www.bioconductor.org), following the methods described by Gentleman et al. [23]. The selection of differentially expressed genes between conditions was based on a linear model analysis with empirical Bayes moderation of the variance estimates [24]. This method approximation is of special interest in microarray data analysis where sample sizes are small. *p* values were adjusted to obtain strong control over the false discovery rate using the Benjamini and Hochberg method [25]. Microarray data have been deposited at ArrayExpress under accession number E-MTAB-3530.

Genes selected as differentially expressed (up- or down-regulated) were classified by their biological processes according to Gene Ontology (GO) database. Determination of biological significance was based on an overrepresentation analysis which establishes whether the differentially expressed genes appeared to be concentrated in GO categories corresponding to specific biological processes. This kind of enrichment tests was also performed using FatiGO from Babelomics tool. Fisher’s exact test for 2 × 2 contingency tables and multiple test correction are applied to find significant overrepresentation of GO terms.

### Morphological analysis of the mammary gland

Dissected right abdominal glands were fixed in 10 % neutral-buffered formalin for 48 h, defatted in acetone, rehydrated and stained in alum carmine. Tissues were dehydrated in graded alcohol, cleared in HistoChoice (Sigma-Aldrich), trimmed of excess of connective tissue under a stereomicroscope and coverslipped with mounting media. Microscopic epithelial proliferative structures were evaluated under a Nikon Eclipse E800 light microscope and the image software ACT-1 for DXM 1200F version 2.51 (Nikon Instruments Europe B.V., Amsterdam, Netherlands). Hyperplastic and neoplastic lesions were identified: hyperplasias, intra-ductal proliferations (IDP) and tumors.

### Apoptosis detection

Tissues were fixed in 4 % phosphate-buffered formalin, paraffin-embedded and cut in 5-µm sections. Apoptosis was assessed through terminal deoxynucleotidyl transferase dUTP nick end labeling (TUNEL) assay using the ApopTag Plus Peroxidase In Situ Detection Kit (Chemicon, Concord Road, MA, USA) according to the manufacturer protocol. Epithelial structures were identified in the mammary glands using the criteria previously established [26] (terminal end buds—TEB, ducts and lobules), and apoptotic cells were visualized under an Axiostar Plus microscope (Carl Zeiss Microscopy, Jena, Germany). To minimize variation, all samples were examined and photographed with the same microscope settings.

### Immunohistochemistry

Infiltration of T cells in the different epithelial structures of the mammary gland was visualized by immunohistochemical detection of CD3-positive cells. Formalin-fixed paraffin-embedded tissue sections were incubated with a specific antibody against CD3 antigen (ab 16669, Abcam, Cambridge, UK) and a biotinylated anti-rabbit secondary IgG antibody (BA-1000, Vector Labs, Burlingame, CA, USA). Immunoreactivity was detected by incubation with chromogen diaminobenzidine (DAB) for 6 min. Tissue sections were counterstained with hematoxylin, dehydrated through graded ethanol and xylene, and cover-slipped. CD3-positive cells were visualized, and images were captured with an Axiostar Plus microscope (Carl Zeiss Microscopy).

### Other statistical analysis

Body weight throughout the assay was analyzed using nonlinear mixed models. We used parametric or nonparametric statistics depending on the distribution of each variable studied, determined by Kolmogorov–Smirnov test, and the equality of variances among groups, determined by Levene’s test. Parametric quantitative data were analyzed with ANOVA followed by Tukey’s test. Analysis of nonparametric quantitative data was performed with Friedman and Mann–Whitney’s *U* tests. Since results along time corresponded to tissues from non-induced (36 and 51 days) and DMBA-induced (100 and 246 days) animals, and most of the data did not follow a normal distribution, in order to homogenize results, nonparametric statistic is represented. Qualitative data were analyzed with Pearson’s Chi-squared test. Differences were considered significant when *p* < 0.05.

## Results

### Effects of high-fat diets on body weights and metabolism

**Fig. 2:**
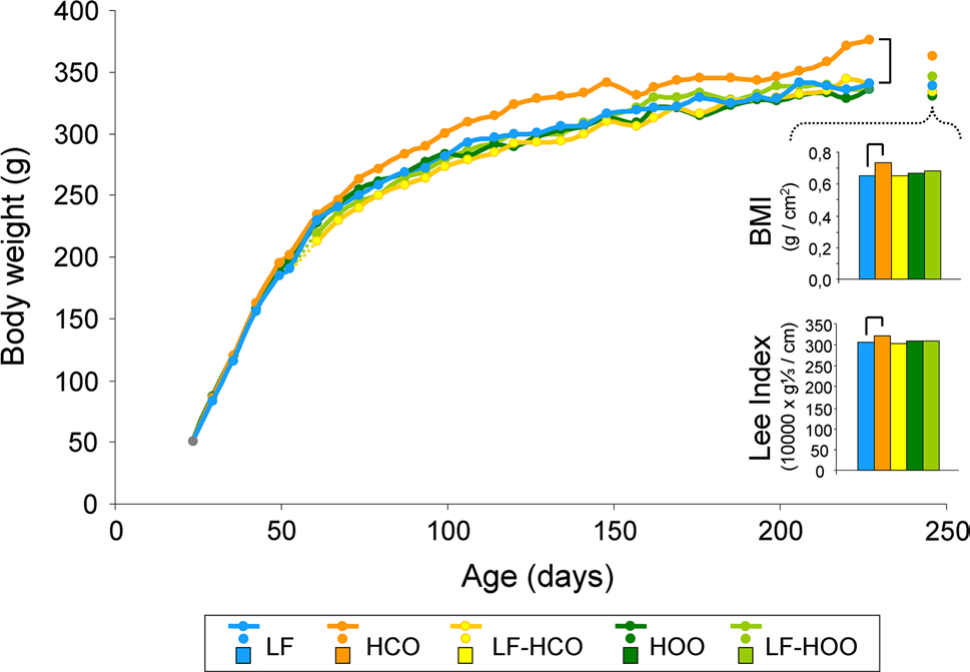
Body-weight evolution and body mass indexes (BMI and Lee index) at the end of the assay (236–256 days, median 246 days). Data represent medians of the groups. For body-weight evolution, number of determinations was as follows: high-fat groups: *N* = 37 (from 24 to 35 days), 31 (from 36 to 50 days), 25 (from 51 to 99 days) and 20 (from 100 onwards); control: *N* = 87 (from 24 to 35 days), 81 (from 36 to 50 days), 75 (from 51 to 53 days), 25 (from 54 to 99 days) and 20 (from 100 onwards). Body mass indexes, *N* = 20/group. Lines connecting groups indicate differences statistically significant (*p* < 0.05), nonlinear mixed models (body weight) and Mann–Whitney’s *U* test (body mass indexes)

Animals fed the high-corn-oil diet from weaning (group HCO, Fig. 1) increased body weight (Fig. 2). In contrast, administration of the high EVOO throughout life (group HOO) did not modify body weight. These high-fat diets, administered after puberty (from induction onwards, i.e., groups LF-HCO and LF-HOO), had no effect on this parameter.

There were no differences in the body mass indexes at 36, 51 and 100 days by effect of the high-fat diets (results not shown). At the end of the assay, body mass index (BMI) and Lee index were increased in HCO group in relation to control LF group (Fig. 2).

**Fig. 3:**
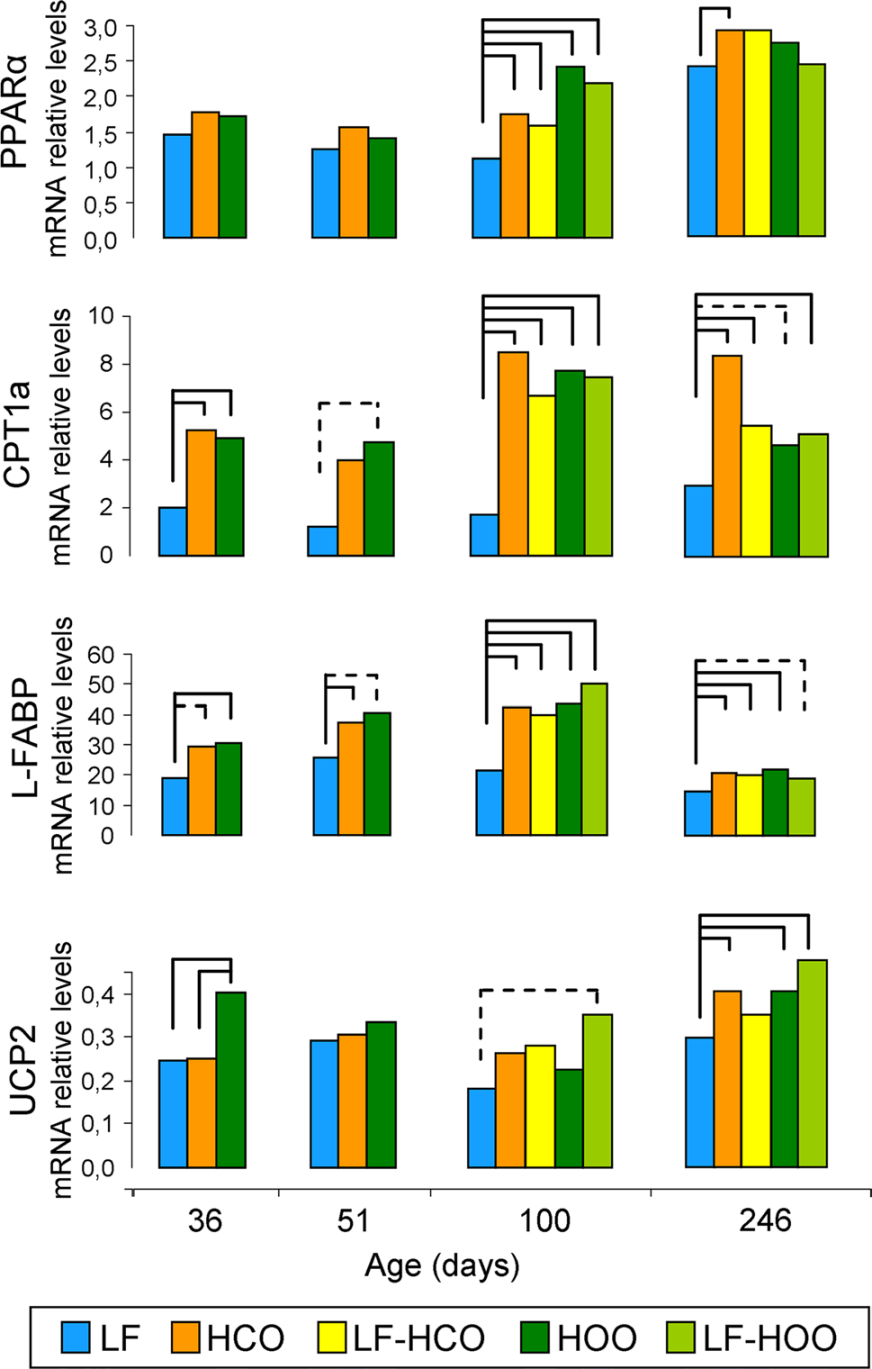
Relative hepatic mRNA expression of genes with a role in lipid transport, metabolism and energy balance. Data shown represent medians of the groups (36 and 51 days *N* = 6/group; 100 days *N* = 5/group, 246 days *N* = 20/group). *Full lines* connecting groups indicate differences statistically significant (*p* < 0.05), and *dotted lines* indicate differences close to significance (*p* < 0.1), Mann–Whitney’s *U* test

High-fat diets modulated the hepatic expression of genes with a role in lipid metabolism and transport. Effects on peroxisome proliferator-activated receptor alpha (PPARα) expression were only evident at 100 days, when all high-fat diet groups increased the mRNA levels of this gene. All high-fat diet groups had also increased expression of carnitine palmitoyltransferase 1a (CPT1a) and liver-type fatty-acid-binding protein (L-FABP) compared to control group at all ages, most of these differences being statistically significant. Uncoupling protein 2 (UCP2) was increased specially by effect of the high EVOO diet, mainly around puberty (36 days; Fig. 3).

### Effects of high-fat diets on gene the expression profile of the mammary gland

**Fig. 4:**
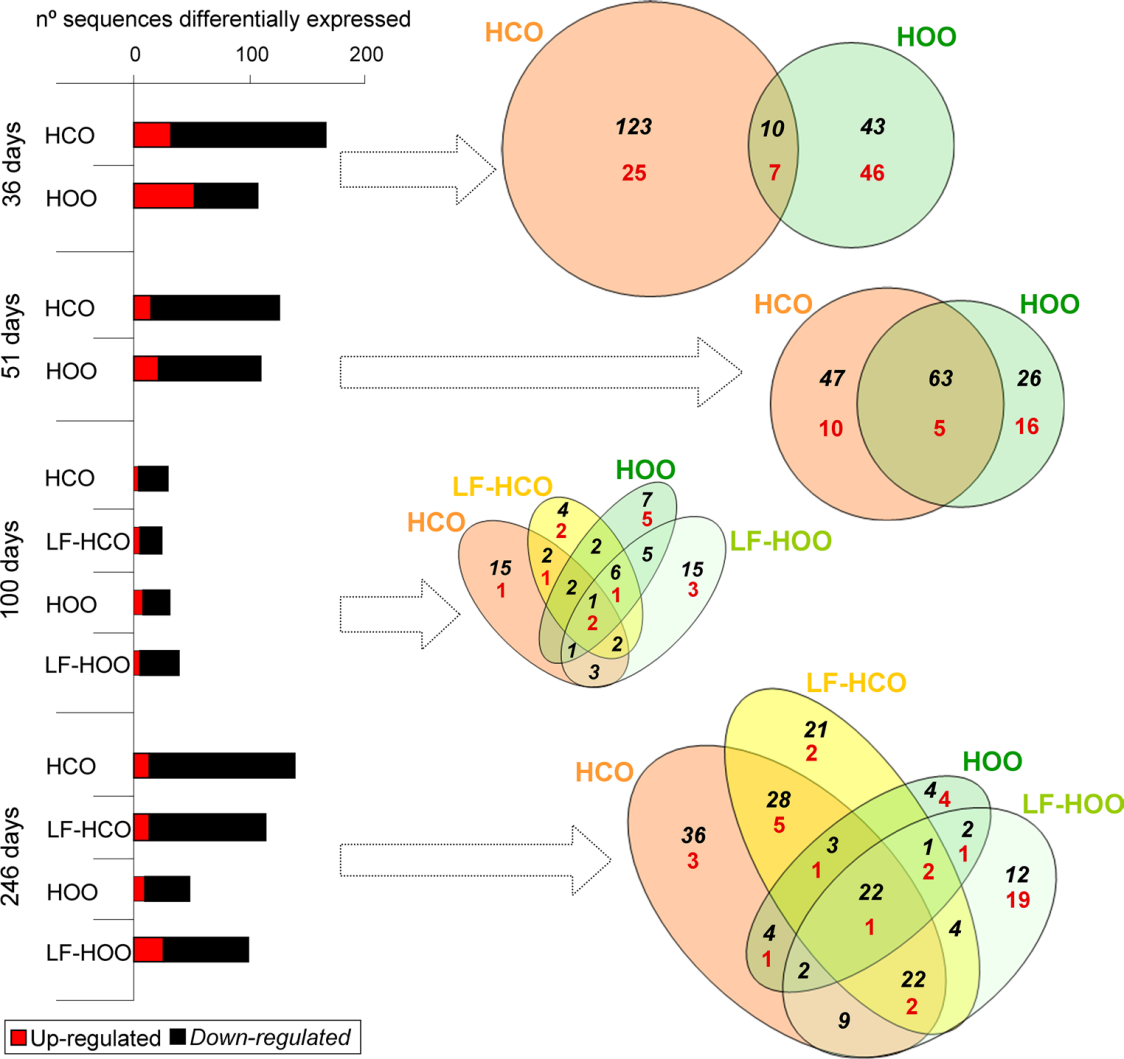
Number of sequences differentially expressed (Benjamini and Hochberg false discovery rate method) in mammary glands of animals fed the high-fat diets (*N* = 3 gene expression profiles/group/age). For each age, Venn diagrams depict overlapping genes within high-fat diet groups being significantly different from low-fat control group. *Black italic numbers* represent down-regulated genes, and* red numbers* represent up-regulated genes

Results of transcriptome analyses in mammary glands from high-fat groups, in comparison with the low-fat control group, showed a larger number of down-regulated genes than up-regulated ones. Total number of modulated genes was higher in tissues from high-corn-oil diet fed animals, except for 100 days, when few differences among groups were observed (Fig. 4). For each age, lists of differentially expressed sequences were compared to identify genes modulated in common (co-modulated by effect of the high-fat diets), partially in common or unique to each specific group. Venn diagrams in Fig. 4 depict number of overlapping and specific genes. At 36 days, most of the modulated genes were specific of the group. On the contrary, at 51 days, we found an important cluster of genes modulated by the effect of both high-fat diets. As already mentioned, few sequences were modulated by 100 days. At the end of the assay (246 days), one cluster of genes corresponded to down-modulated in all high-fat diet groups. Moreover, a significant number of genes were modulated by effect of the high-corn-oil diet (in both HCO and LF-HCO groups, or specifically in each one) but not by the high EVOO diet.

Functional clustering of differentially expressed genes resulted in significantly enriched Gene Ontology (GO) categories corresponding to biological processes. Enriched categories for all genes found in each group (specific or common) are detailed in Supplementary Table 2. Among the down-modulated genes, some biological processes were generally found in several groups and at different ages, such as metabolism and response to stimulus. Thirty-six days was the age with more dissimilarities among groups, when genes down-regulated in HCO group were related to immune response, cell death, proliferation and adhesion. Cell functions such as proliferation and cell death were also overrepresented in down-modulated genes at different ages. Functional analysis was also performed dividing commonly modulated genes and specific for each group. Extended enriched categories are detailed in Supplementary Table 3 (36 days of age), Supplementary Table 4 (51 days of age) and Supplementary Table 5 (246 days of age). No significant enriched categories were found at 100 days when modulated genes were divided in specific and common. Results revealed different trends in groups fed the high-corn-oil diet and in the ones fed the high olive oil diet. Common genes and genes specifically modulated in the high EVOO groups were mainly related to metabolic processes. On the other hand, genes down-modulated by effect of the high-corn-oil diet had a role on apoptosis and immune system, mainly at 36 days of age.

**Fig. 5:**
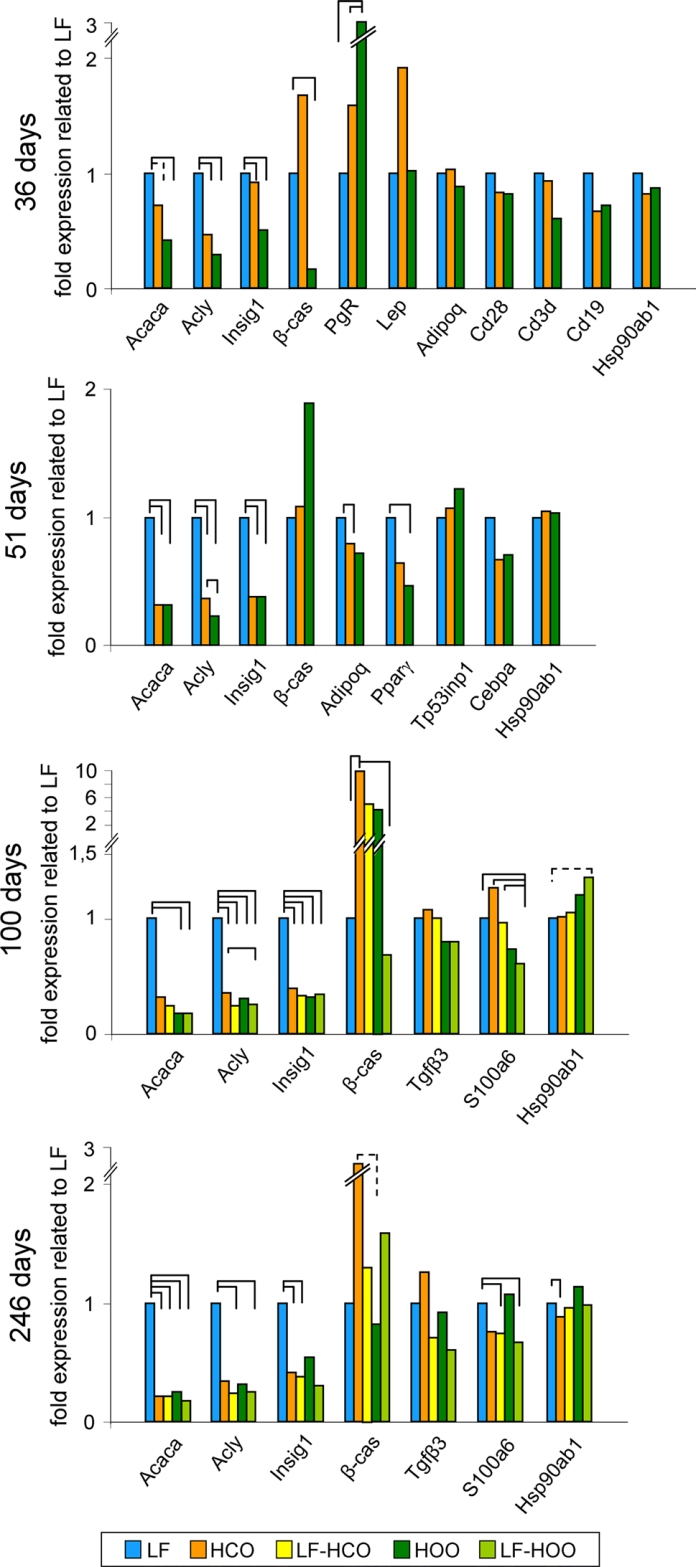
Real-time PCR validation of genes found as modulated in the microarrays analysis. Data represent relative gene expression (median) (36 and 51 days *N* = 6/group; 100 days *N* = 5/group, 246 days *N* = 20/group). *Full lines connecting groups* indicate statistically significant differences (*p* < 0.05), and *dotted lines* indicate differences close to significance (*p* < 0.1), Mann–Whitney’s *U* test

**Fig. 6:**
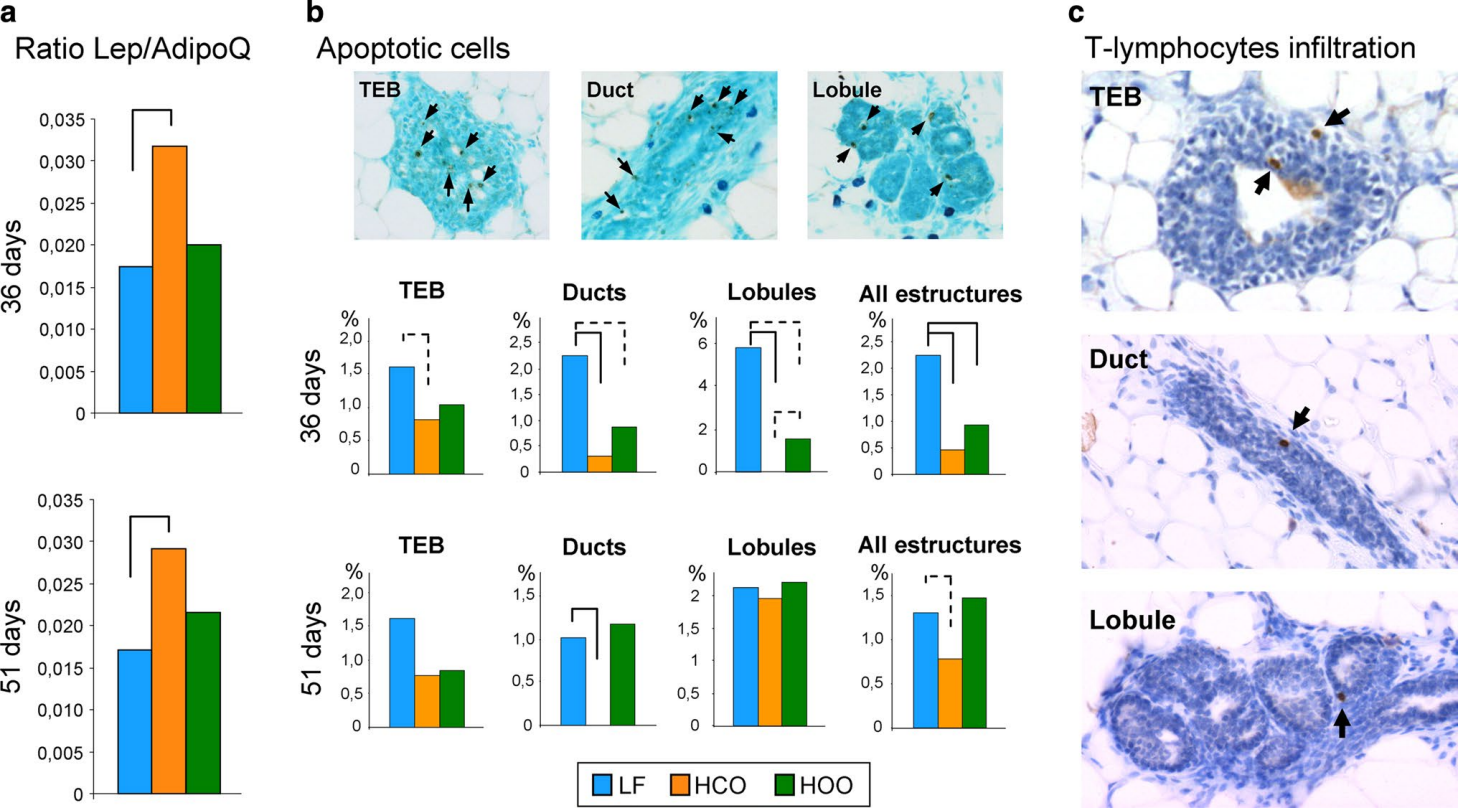
Ratio between leptin and adiponectin expression (Lep/AdipoQ) in mammary glands of the experimental groups at peri-pubertal ages (*N* = 6/group) (**a**). Detection of apoptosis in mammary epithelial structures and percentage of apoptotic cells at 36 and 51 days in terminal end buds (TEB), ducts, lobules, as well as considering all structures (*N* = 8–21 each structure/group/age);* arrows* indicate positive cells (**b**). Detection of T cells by immunohistochemistry in TEB, ducts and lobules; *arrows* indicate positive cells (**c**). *Full lines connecting groups* indicate statistically significant differences (*p* < 0.05), and *dotted lines* indicate differences close to significance (*p* < 0.1), Mann–Whitney’s *U* test

We further selected for validation several genes related to overrepresented processes at different ages. Results of gene expression analyzed by real-time PCR are shown in Fig. 5. Genes related to lipid synthesis pathways (acetyl-CoA carboxylase alpha—Acaca, ATP citrate lyase—Acly, insulin-induced gene 1—Insig1) were significantly down-modulated by the effect of both high-fat diet at all ages tested. mRNA expression of genes with a role in mammary gland morphogenesis was also modulated at different ages. Thus, β-casein (β-cas) was in general up-modulated in HCO group, and progesterone receptor (PgR) was up-modulated in HOO group at 36 days of age. Lepin (Lep) tended to be up-modulated at 36 days in HCO, and adiponectin (AdipoQ) was down-modulated in the same group at 51 days. Due to the reported importance of the ratio Lep/AdipoQ [27], analyses of these genes were extended to all ages (Fig. 6a). Such ratio was significantly increased in HCO at 36 and 51 days of ages. On the other hand, differences in the expression of genes related to immune function (Cd28, Cd3d, Cd19) by high-fat diets were not validated. Moreover, expression of genes with a role in cell death (Tp53inp1 and Cebpa) showed different trends in HCO and HOO at 51 days, but results did not reach statistical significance. Finally, the mRNA levels of genes with a role in response to stimulus (Tgfβ3, S100a6, Hsp90ab1) also showed different trends in HCO and HOO groups at 100 and 246 days, specially lower levels of S100a6 in LF-HOO (100 days) and lower levels of Hsp90ab1 in HCO (246 days).

### Effects of high-fat diets on apoptosis and T cell infiltration

In order to further investigate the effects of the high-fat diets on the expression of genes with a role in functions that may modify breast cancer susceptibility, immunohistochemical analyses of paraffin-embedded tissue were performed. As already mentioned, real-time PCR analysis in relation to genes with a role in apoptosis showed different trends in HCO and HOO groups, but results did not reach statistical significance. Detection of cell death by TUNEL showed a lower percentage of apoptotic cells in the mammary glands of animals fed the high-corn-oil diet at 36 and 51 days and tended to be lower in the glands of the animals fed the high EVOO diet at 36 days. Such effect was observed in different epithelial structures: terminal end buds (TEB), ducts, lobules and considering all of them (Fig. 6b). These effects of the diets were observed at peri-pubertal ages, but not in adulthood (results not shown). Moreover, we assessed by immunohistochemistry the infiltration of T lymphocytes by detection of CD3 marker (Fig. 6c). We detected few T cells in TEB, ducts and lobules, and no significant differences were observed by effect of the high-fat diets (results not shown).

### Effects of high-fat diets on carcinogenesis

**Fig. 7:**
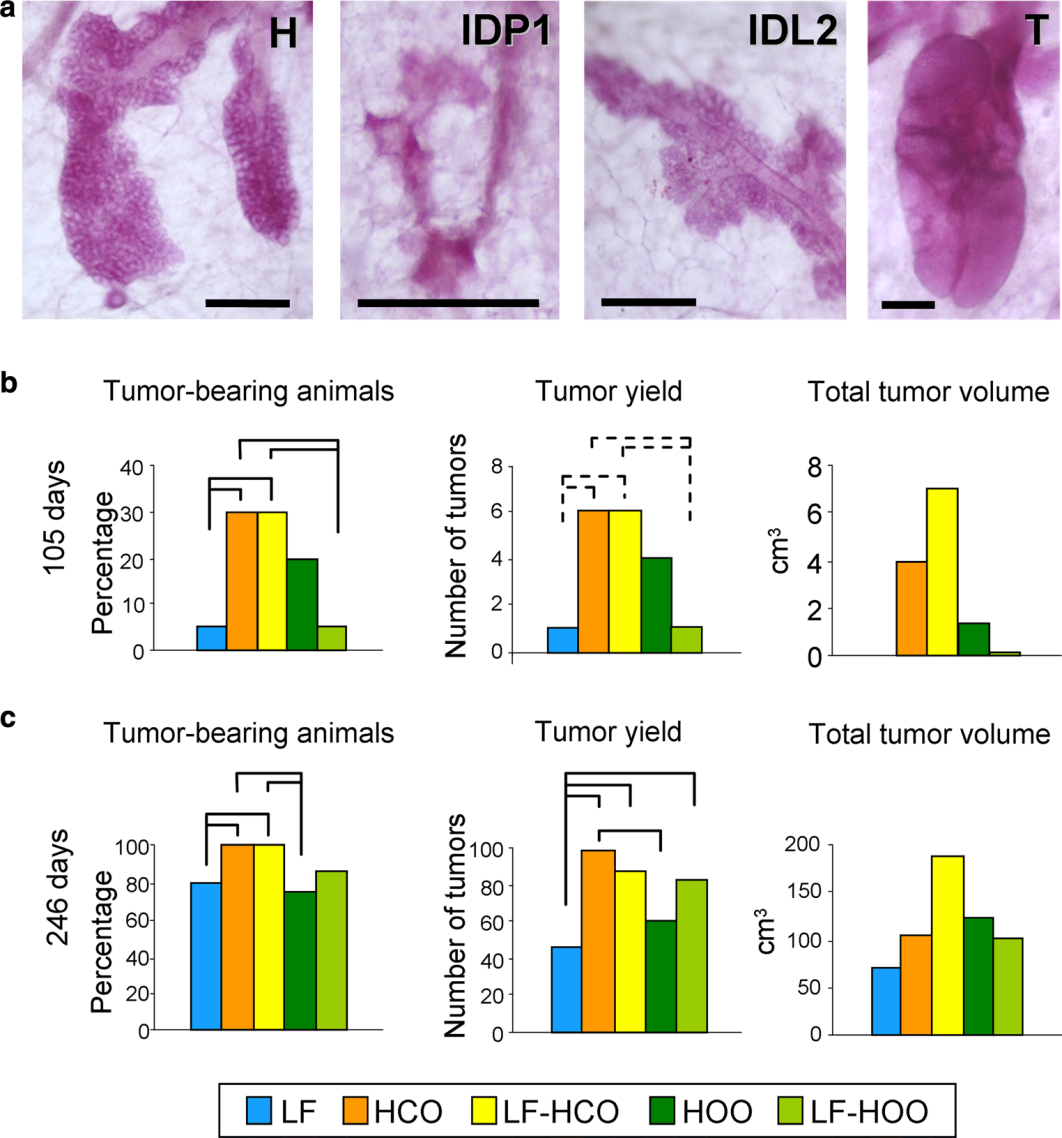
Epithelial proliferative lesions in whole-mounted mammary glands: hyperplasia (H), small intra-ductal proliferation (IDP1), large intra-ductal proliferation (IDP2), tumor (T); scale bar 250 μm (**a**). Carcinogenesis parameters at 105 days of age (52 days after DMBA induction, *N* = 20/group) (**b**). Carcinogenesis parameters at the end of the assay (236–256 days of age, *N* = 20/group) (**c**). *Full lines connecting groups* indicate statistically significant differences (*p* < 0.05); *dotted lines* indicate differences close to significance (*p* < 0.1), Mann–Whitney’s *U* test

Microscopic proliferative lesions were identified in the whole-mounted mammary glands: hyperplasias, small intraductal proliferation (IDP1), large intraductal proliferation (IDP2) and tumors (Fig. 7a). At 100 days of age (47 days after DMBA induction), no hyperplasias were found: one IDP1 in group LF-HCO, two IDP2 in groups HCO and HOO (1 each) and three tumors in group HCO. At the end of the assay, all type of structures were found in all groups, but most of the mammary glands were damaged during the removal of the embedded tumors and whole-mounted glands were technically difficult to analyze (results not shown).

On the other hand, clinical manifestation of cancer was studied by determination of carcinogenesis parameters. At 52 days after DMBA induction (105 days of age), the carcinogenesis yield was low (Fig. 7b). At that age, tumor-bearing animals and total tumor yield were increased in both high-corn-oil diet groups (HCO and LF-HCO), in relation to LF-HOO and control groups. Groups fed the high-corn-oil diet had also the highest value of total tumor volume, but no statistical analysis can be performed since this is a quantitative parameter with only one value per group. At the end of the assay, all the carcinogenesis parameters were increased in all groups (Fig. 7c). Groups of animals fed the corn oil-enriched diet showed the highest percentage of tumor-bearing animals, significantly different in comparison with control and HOO groups. Total number of tumors was increased in HCO (significantly higher values than control and HOO), followed by LF-HCO and LF-HOO (increased compared to control). Finally, LF-HCO had also at that age the highest value of total tumor volume (Fig. 7c).

## Discussion

We have previously reported that high-fat intake influence DMBA-induced breast cancer susceptibility [3, 4, 28, 29]. In this work, we have investigated whether diets rich in corn oil or in extra-virgin olive oil have a different influence on experimental mammary carcinogenesis through a differential effect on development (including body growth and metabolism), also affecting the molecular context of the mammary gland. The high-corn-oil diet, when administered from weaning, increased body weight and mass, but the high EVOO diet did not, probably related to an increase in energy expenditure through mitochondrial uncoupling proteins. These diets have different effects on mammary gland transcriptome profile, especially short after dietary intervention, with the high-corn-oil diet down-regulating genes related to immune system and apoptosis. Further immunohistochemical investigation suggested a decrease in apoptosis in the mammary epithelial structures of animals fed the high-corn-oil diet, what can be one of the mechanisms of its clear stimulating influence on mammary carcinogenesis.

Nutritional factors influencing growth, maturation and adiposity may have an impact on the susceptibility to mammary transformation [2]. Actually, obesity and early menarche are well-known risk factors for human breast cancer [2, 12]. Here we have observed a different influence of the high-corn-oil and EVOO diets on body weight when administered from weaning, although no changes were evident if diets were administered after puberty (when the carcinogenesis has been already initiated). Hence, the HCO group, but not the HOO one, had higher body weight throughout the study and body mass at the end of the assay. Considering that both high-fat diets have an excess in lipid content, these data highlight that not only the caloric intake but also the type of lipid consumed are important factors in the control of body weight. The results obtained suggest that these two types of lipid may have different effects on energy intake/expenditure balance, and are also in accordance with other authors reporting that olive oil produce less body-weight gain than saturated fat [30] and corn oil [31]. Thus, we further analyzed whether these diets have an effect on the hepatic expression of genes with a role in energy balance, lipid transport and metabolism, such as PPARα and its target genes CPT1a, L-FABP and UCP2. A different effect was only observed in UCP2, up-regulated mainly by effect of the high EVOO diet. In accordance with this, an olive oil diet has also been reported to increase the expression of UCPs in brown adipose tissue and muscle [31, 32]. UCPs are mitochondrial proteins uncoupling oxidative phosphorylation and therefore dissipating energy as heat. All together, these results suggest an increase in liver lipid uptake and β-oxidation by effect of both high-fat diets [33], probably related to the physiological processing of lipids, but an increase in energy dissipation (thus decreasing the ratio energy intake/expenditure) only by effect of the high EVOO diet. Although caution must be applied when comparing human data and animal models, these results are in accordance with epidemiological data showing that Mediterranean diet, with the main source of fat being olive oil, has a protective effect on body weigh gain and obesity [10, 11, 34].

Changes in body growth, sexual maturation and energy intake/expenditure balance may influence breast cancer susceptibility [2, 12]. Aiming to investigate molecular mechanisms driving the effects of the experimental diets, we determined the gene expression profile of the mammary gland at 36, 51, 100 and 246 days of age. These ages are of special interest to study the susceptibility to malignant transformation and the promotion of cancer. At 36 days, just after puberty onset, the mammary gland is actively developing, period which extends to 51 days. Moreover, this time point (51 days) is of particular relevance as it is within the window of maximum susceptibility to malignant transformation [35], 2 days before the induction with DMBA. After induction, around 100 days of age (47 days post-DMBA) carcinogenesis begins to clinically manifest in all groups. With the DMBA dose used (5 mg), in order to study the promotion of cancer, the assays are typically extended to 200–250 days.

Transcriptomic analysis in mammary glands showed different effects of corn oil and extra-virgin olive oil. In general, the high-n-6 PUFA diet regulated a larger number of sequences. By 36 days of age, the diets induced a clear specific gene expression signature, and few genes were commonly modulated by both diets. The total number of modulated sequences decreased thereafter and an important set of genes were commonly modulated (co-regulated by the high-fat diets). Functional clustering of modulated genes resulted in significantly enriched categories showing different trends also depending on the diet. At different ages tested, co-regulated genes and genes modulated only in EVOO groups were frequently related to metabolism. Genes only down-modulated by the corn oil-based diet were recurrently involved in immune system and apoptosis, functions related to cancer susceptibility [36, 37]. At the light of those results, the effects of high-fat diets seem to be stronger and more specific after a short period (at 36 days, 12 days after beginning of dietary intervention) than after longer administration, and both diets induce different changes in the mammary gland. In rat colonic mucosa, it has also been described specific gene expression signatures depending on the type of lipid intake (fish oil, corn oil or olive oil [38]).

Validation analysis by real-time PCR confirmed the co-modulation of genes with a role in lipid metabolism, specially the inhibition of lipid synthesis, as a response to the high lipid intake that extends to the adaptation to chronic consumption. Both high-fat diets clearly down-regulated the rate-limiting enzyme in fatty acid synthesis (Acaca), the enzyme for the synthesis of cytosolic acetyl-CoA (Acly) and the key enzyme that regulates cholesterol concentrations (Insig1). We also observed changes in β-casein and progesterone receptor at puberty that were concordant with the advanced puberty reported [19], and an increase in β-casein in adulthood compatible with the higher degree in gland density in the HCO group previously observed [19]. Regarding the adipocytokines, leptin and adiponectin, they have been reported to exert antagonistic effects on mammary gland development [27, 39]. Thus, we calculated the ratio leptin/adiponectin finding a significant increase by effect of the corn oil-enriched diet at peri-pubertal ages, what is compatible with a more proliferative gland [27] just before DMBA induction. On the other hand, results for immune system genes showed a great variability among samples, and differences found in the transcriptomic analysis were not validated. Since most of the immune system genes found in the microarrays were lymphocyte T markers, immunohistochemical detection of T cells was carried out, observing few cells in the different epithelial structures. Intra-epithelial T cells have been identified in the normal human breast, but little is known about their specific function [40]. As there is hormonal control of the immune system that may contribute to mammary development via recruitment of immune cells [40], the lack of evident changes in immune system genes may be related to sample collection at different stages of the estrus cycle.

An important cluster of genes differentially regulated by the high-fat diets was the one related to apoptosis. Thus, by 51 days of age, results suggested a decrease in apoptosis genes such as CCAAT/enhancer binding protein alpha (Cebpa) and tumor protein p53 inducible nuclear protein 1 (Tp53inp1) by effect of the n-6 PUFA-enriched diet. Cebpa is regulated by adiponectin, and both were down-regulated in the mammary glands of HCO group. Both Cebpa and Tp53inp1 can be down-regulated in breast cancer [41, 42], and although validation also suggested a different effect of those diets in their expression, evident differences were not obtained. However, further histological detection of apoptotic cells by TUNEL confirmed a decrease in apoptosis in the epithelial cells in HCO group. These data suggested that changes in the apoptotic capacity of the mammary gland may be one of the molecular mechanisms by which the corn oil-enriched diet has a clear stimulating effect on mammary carcinogenesis.

In the DMBA-induced adult mammary gland, other genes also showed different trends depending on the diet administered, such as Tgfβ3 and Hsp90ab1, but differences did not reach statistical significance. Finally, glands from LF-HOO group had down-regulated expression of S100a6 at both adult ages tested. S100 genes have a role in breast cancer progression, and it has been reported a decrease in S100a6 concomitantly with a reduction in oxidative stress [43]. These data are in accordance with lower cancer-promoting microenvironment in the glands of rats fed the olive oil-enriched diet and can be related to the different carcinogenesis evolution observed among groups. In this sense, in mice, a high-fat diet also increased mammary cancer susceptibility and elevated the inflammatory gland environment [44].

The experimental diets have also exerted different effects on the clinical evolution of the cancer disease. In accordance with previous results [3, 4, 28, 29], the diet rich in n-6 PUFA had a clear stimulating effect on breast carcinogenesis. At day 52 after DMBA induction (105 days of age), the carcinogenesis yield was low in all groups. The study of microscopic proliferative structures showed more epithelial lesions (mainly microtumors) in groups fed the diet rich in n-6 PUFA. Clinical parameters of the carcinogenesis had also higher values in such groups, while results from the olive oil fed groups had no significant differences compared to controls. At 246 days, the carcinogenesis yield was higher in all groups and values were more similar among them, which suggests that in the long term, the progression of cancer disease ends up being stronger than the action of environmental factors. Nevertheless, there was also a clear stimulating effect of the diet rich in n-6 PUFA, whereas the groups fed the high EVOO diet were more similar to the low-fat control. There is human and experimental evidence that total intake of fat positively correlates with breast cancer risk [2, 45]. Hence, while the stimulating effect of the high-corn-oil diet is clear and strong, the more variable and weaker influence of the high EVOO diet, despite being high fat, suggests that this type of oil has some beneficial effect that may partially counteract the total fat intake. Several active components of olive oil have been suggested to have health benefits, including the MUFA oleic acid or the phenolic minor compounds [46, 47]. We previously reported in experimental mammary tumors that these two types of diet exert a different modulation of cell-signaling pathways that modifies the cell proliferation–apoptosis balance, showing higher proliferation by effect of the high corn oil and higher apoptosis by the high EVOO [48]. Results obtained in this work also suggest a different influence of these diets on breast cancer susceptibility leading a different proliferation–apoptosis balance in the pubertal mammary gland as well and inducing a different promoting microenvironment.

The importance of the high-fat intake before puberty on breast cancer susceptibility has also been reported by other authors. Prepubertal exposure to a low-n-3 PUFA diet in rat reduced cell proliferation, increased apoptosis and lowered breast cancer risk in adulthood, whereas a prepubertal high-n-3 PUFA intake had opposite effects [49]. In BALB/c mice, a commercial diet rich in high fat (from corn oil and lard) induced increased mammary epithelial cell proliferation, macrophage infiltration, angiogenesis and inflammatory factors [44]. Here we have observed a different effect of diets enriched in corn oil or EVOO on peri-pubertal mammary gland compatible with an increase in susceptibility by effect of high-corn-oil intake during puberty. However, this type of oil has shown a strong promoting effect eliciting that, even if the susceptibility to malignant transformation is increased, the fast growth and progression of carcinogenesis in the animals fed that diet made no evident clinical difference upon the beginning of dietary intervention (childhood or adulthood, e.g., group HCO or LF-HOO). The effect of the high EVOO diet on susceptibility seemed to be weaker, and carcinogenesis from animals fed this diet progressed more slowly (in the long term) if dietary exposure began in childhood than in adulthood. Taken together, all those results highlight the complex and sometimes subtle influence that dietary factors may have on initiation, promotion and progression of breast tumors, likely inducing diverse molecular contexts (in the tumor and in the adjacent mammary gland) that differentially influence each carcinogenesis stage.

## Conclusions

The data obtained in this work suggest that the high-corn-oil and EVOO diets, when administered from an early age, elicit different growth and long-term adiposity, with the high-corn-oil diet increasing body mass index and the olive oil-enriched one avoiding, at least in part, lipid storage thought modulation of mitochondrial uncoupling proteins. The dietary exposure also induced specific gene expression profile in the mammary glands, suggesting a more disrupted signature by effect of the high corn oil than by the EVOO diet. The effect of the one high in corn oil is compatible with an increase in proliferation/apoptosis balance in the mammary gland, which could be one of the mechanisms of the increased susceptibility to malignant transformation and the promoting microenvironment in this tissue. These data would be in accordance with the morphological transformation and the clinical manifestation of the carcinogenesis, clearly stimulated by the diet rich in PUFA n-6, and with a weaker effect of the EVOO diet. Taken together, the results point out the importance of dietetic habits, especially (but not exclusively) from early ages, on future breast cancer risk.

## Electronic supplementary material

Below is the link to the electronic supplementary material.

Supplementary Table 1 Composition of the experimental diets (PDF 19 kb)

Supplementary Table 2 Enriched Gene Ontology (GO) categories in down- and up-modulated genes in mammary gland by effect of the high-corn-oil and the high-extra-virgin olive oil diets at 36, 51, 100 and 246 days of age (PDF 53 kb)

Supplementary Table 3 Enriched Gene Ontology (GO) categories in commonly or specifically modulated genes in mammary gland by effect of the high-fat diets at 36 days. Down- and up-modulated genes in each group were sub-classified in “common” (modulated in more than one group) or “specific” (found as modulated in one specific group) (PDF 64 kb)

Supplementary Table 4 Enriched Gene Ontology (GO) categories in commonly or specifically modulated genes in mammary gland by effect of the high-fat diets at 51 days. Down- and up-modulated genes in each group were sub-classified in “common” (modulated in more than one group) or “specific” (found as modulated in one specific group) (PDF 48 kb)

Supplementary Table 5 Enriched Gene Ontology (GO) categories in commonly or specifically modulated genes in mammary gland by effect of the high-fat diets at 246 days. Down- and up-modulated genes in each group were sub-classified in “common” (modulated in more than one group) or “specific” (found as modulated in one specific group) (PDF 37 kb)
