# Supplementary material for: Diets high in corn oil or extra-virgin olive oil differentially modify the gene expression profile of the mammary gland and influence experimental breast cancer susceptibility
Source: Eur J Nutr. 2015 Jun 20;55:1397–409. doi: 10.1007/s00394-015-0958-2 (PMC4875377; doi:10.1007/s00394-015-0958-2)
Supplement: Supplementary file 1 — Composition of the experimental diets (PDF 19 kb) [file 394_2015_958_MOESM1_ESM.pdf]

Article title: Diets high in corn oil or extra virgin olive oil differentially modify the gene expression profile of the mammary gland and influence experimental breast cancer susceptibility

Journal name: European Journal of Nutrition

Authors names: Raquel Moral[1], Raquel Escrich[1], Montserrat Solanas[1], Elena Vela[1], M. Carme Ruíz de Villa[2], Eduard Escrich[1].

Affiliations: [1]Department of Cell Biology, Physiology and Immunology, Physiology Unit, Medicine School, Universitat Autònoma de Barcelona, 08193 Bellaterra, Barcelona, Spain; [2]Department of Statistics, Universitat de Barcelona, 08028 Barcelona, Spain.

Corresponding author: Dr. Eduard Escrich; Department of Cell Biology, Physiology and Immunology; Physiology Unit, Medicine School; Universitat Autònoma de Barcelona; 08193 Bellaterra (Barcelona), Spain. Phone: +34 93 581 14 23, Fax: +34 93 581 29 86. Eduard.Escrich@uab.es

Electronic Supplementary Material 1:

Supplementary Table 1

Composition of the experimental diets

Diet composition (g/kg)	Low fat	High corn oil	High olive oil
Proteins (casein)	180	230	230
Carbohydrates (dextrose)	679	459	459
Lipids:			
Corn oil	30	200	30
Extra virgin olive oil	0	0	170
Fibre (cellulose)	50	50	50
Mineral mix[a]	59	59	59
Vitamin mix[a]	2.4	2.4	2.4

Fatty acids in the oils (%)	Corn oil	Extra-virgin olive oil
C16:0 (palmitic)	11.1	13.2
C16:1 (palmitoleic)	0.2	1.3
C17:0 (margaric)	0.1	0.1
C17:1 (margaroleic)	0.0	0.2
C18:0 (stearic)	2.3	2.5
C18:1 (oleic)	31.7	73.7
C18:1 *trans-double bound* (elaidic)	0.1	0.0
C18:2 (linoleic)	51.3	7.3
C18:2 *trans-double bound* linoleics	1.3	0.0
C18:3 (linolenic)	0.5	0.8
C18:3 *trans-double bound* linolenics	0.3	0.0
C20:0 (arachidic)	0.5	0.4
C20:1 (eicosenoic)	0.2	0.3
C22:0 (docosanoic)	0.2	0.1
C24:0 (lignoceric)	0.2	0.1
Total saturated	14.4	16.4
Total monounsaturated	32.1	75.5
Total n-6 polyunsaturated	51.3	7.3
Total n-3 polyunsaturated	0.5	0.8
Total *trans-double bound* isomers	1.7	0.0

[a]: The composition of mineral and vitamin mixes is given in [1]

[1] Escrich E, Solanas M, Segura R (1994) Experimental diets for the study of lipid influence on induced mammary carcinoma in rats: I – Diet definition. In Vivo 8: 1099-1106
