# Supplementary material for: Diets high in corn oil or extra-virgin olive oil differentially modify the gene expression profile of the mammary gland and influence experimental breast cancer susceptibility
Source: Eur J Nutr. 2015 Jun 20;55:1397–409. doi: 10.1007/s00394-015-0958-2 (PMC4875377; doi:10.1007/s00394-015-0958-2)
Supplement: Supplementary file 2 — Enriched Gene Ontology (GO) categories in down- and up-modulated genes in mammary gland by effect of the high-corn-oil and the high-extra-virgin olive oil diets at 36, 51, 100 and 246 days of age (PDF 53 kb) [file 394_2015_958_MOESM2_ESM.pdf]

Article title: Diets high in corn oil or extra virgin olive oil differentially modify the gene expression profile of the mammary gland and influence experimental breast cancer susceptibility

Journal name: European Journal of Nutrition

Authors names: Raquel Moral[1], Raquel Escrich[1], Montserrat Solanas[1], Elena Vela[1], M. Carme Ruíz de Villa[2], Eduard Escrich[1].

Affiliations: [1]Department of Cell Biology, Physiology and Immunology, Physiology Unit, Medicine School, Universitat Autònoma de Barcelona, 08193 Bellaterra, Barcelona, Spain; [2]Department of Statistics, Universitat de Barcelona, 08028 Barcelona, Spain.

Corresponding author: Dr. Eduard Escrich; Department of Cell Biology, Physiology and Immunology; Physiology Unit, Medicine School; Universitat Autònoma de Barcelona; 08193 Bellaterra (Barcelona), Spain. Phone: +34 93 581 14 23, Fax: +34 93 581 29 86. Eduard.Escrich@uab.es

Electronic Supplementary Material 2:

Supplementary Table 2

Enriched Gene Ontology (GO) categories in down- and up-modulated genes in mammary gland by effect of the experimental high fat diets.

Lists of all genes found modulated in each group were analyzed using Genecodis program by Babelomics platform setting GO annotations at level 3. Only statistically significant enriched categories are indicated.

36 days

HCO down-regulated

Gene Ontology category (level 3)	*Items[a]*	*S[b]*	*TS[c]*	*Hyp[d]*	*Genes[e]*
immune response	GO:0006955	21(154)	305(22938)	1, 65E-10	LOC688090, RT1-Bb, Cd79b, Cd37, Cd74, Hla-dmb, Cd19, ccl21b, hla-dma, Il2rg, Irf8, Ets1, Rab27a, Arbp, Cd28, Lyn, Tgfb1, RT1-Ba, cd8a, Rbm4, Rbm4b
cell activation	GO:0001775	14(154)	119(22938)	6, 69E-09	cd5, hla-dma, Il2rg, Coro1a, Satb1, cd3d, Ets1, Rab27a, Cd28, Cd38, Lyn, Tgfb1, RT1-Ba, cd8a
response to external stimulus	GO:0009605	19(154)	394(22938)	2, 15E-06	Rac2, Coro1a, Satb1, Ptpn6, Ets1, Rab27a, Arbp, Cd28, Ninj1, Ptk2b, Cd38, Sod1, Lyn, Tgfb1, Id3, Ddt, cd8a, Rbm4, Rbm4b
cell adhesion	GO:0007155	9(154)	276(22938)	1, 07E-04	Fnbp4, Selplg, Lef1, Coro1a, Gpnmb, Ets1, Ninj1, Ptk2b, Tgfb1
anatomical structure development	GO:0048856	23(154)	798(22938)	3, 95E-04	Cdc42se1, Nme2, Lef1, hla-dma, Dnase2a, Il2rg, Coro1a, Irf8, Satb1, Gpnmb, LOC364236, cd3d, Ets1, Cd28, Ninj1, Ptk2b, Sod1, Lyn, Tgfb1, Id3, RT1-Ba, Adrbk1, cd8a
response to abiotic stimulus	GO:0009628	7(154)	206(22938)	4, 96E-04	Ets1, Arbp, Ptk2b, Sod1, Lyn, Tgfb1, Rbm4
catabolic process	GO:0009056	13(154)	684(22938)	7, 34E-04	Lyc2, Dnase1l3, Pla2g2d, Atp5o, Abcg1, Arrb2, Nedd8, Phf11, Dnase2a, Lyz2, Ets1, Sod1, Tgfb1
organelle organization	GO:0006996	11(154)	525(22938)	8, 63E-04	Npm1, Tuba1b, Dnase1l3, Timm13, Rac2, Coro1a, Satb1, Ets1, Cd28, Ptk2b, Sod1
microtubule-based process	GO:0007017	5(154)	111(22938)	9, 25E-04	Tuba1a, Tuba1c, Npm1, Tuba1b, Ets1
cell projection organization	GO:0030030	5(154)	133(22938)	2, 06E-03	Rac2, Coro1a, Ets1, Ptk2b, Sod1
response to stress	GO:0006950	21(154)	742(22938)	2, 74E-03	Lyz2, Ptpn6, Ets1, Rab27a, Arbp, Cd28, Ninj1, Ptk2b, Cd38, Sod1, Lyn, Tgfb1, Id3, RT1-Ba, Ddt, Adrbk1, cd8a, Ucp2, Tp53inp1, Rbm4, Rbm4b
actin filament-based process	GO:0030029	4(154)	90(22938)	3, 19E-03	Rac2, Coro1a, Ets1, Ptk2b
response to biotic stimulus	GO:0009607	5(154)	156(22938)	4, 10E-03	Mx2, Mx1, Lyz2, Ets1, Tgfb1
cell proliferation	GO:0008283	13(154)	275(22938)	4, 63E-03	S100a6, cd5, Npm1, Rac2, Coro1a, Satb1, Ets1, Cd28, Ptk2b, Cd38, Lyn, Tgfb1, Id3
behavior	GO:0007610	5(154)	166(22938)	5, 32E-03	Rac2, Coro1a, Ets1, Sod1, Tgfb1
multicellular organismal development	GO:0007275	22(154)	867(22938)	8, 50E-03	Nme2, Lef1, hla-dma, Dnase2a, Il2rg, Coro1a, Irf8, Satb1, Gpnmb, LOC364236, cd3d, Ets1, Cd28, Ninj1, Ptk2b, Sod1, Lyn, Tgfb1, Id3, RT1-Ba, Adrbk1, cd8a
cell death	GO:0008219	14(154)	352(22938)	1, 18E-02	Diablo, cd5, Npm1, Dnase1l3, Dnase2a, Lyz2, Ets1, Rab27a, Cd28, Ptk2b, Sod1, Tgfb1, Id3, Tp53inp1
cellular component movement	GO:0006928	5(154)	220(22938)	1, 66E-02	Selplg, Coro1a, Ets1, Ptk2b, Tgfb1
cellular response to stimulus	GO:0051716	5(154)	250(22938)	2, 70E-02	Coro1a, Ets1, Ptk2b, Sod1, Lyn
sexual reproduction	GO:0019953	4(154)	184(22938)	3, 57E-02	Ets1, Ptk2b, Sod1, Tgfb1

HCO up-regulated

Gene Ontology category (level 3)	*Items*	*S*	*TS*	*Hyp*	*Genes*
multicellular organism growth	GO:0035264	3(33)	88(22938)	2, 74E-04	Igf1, Tshr, Lep
response to external stimulus	GO:0009605	5(33)	914(22938)	9, 33E-03	F13a1, Serpina3n, Alb, Igf1, Lep
behavior	GO:0007610	7(33)	443(22938)	2, 64E-02	Alb, S100b, Igf1, Tshr, Oxtr, Lep, Sncg

HOO down-regulated

Gene Ontology category (level 3)	*Items*	*S*	*TS*	*Hyp*	*Genes*
alcohol metabolic process	GO:0006066	14(67)	546(22938)	4, 89E-05	Pfkfb3, Bche, Tkt, Pfkl, Taldo1, Pygl, Pdk1, Insig1, Chpt1, Pc, Cyb5r3, Dhcr7, Pdhb, pdha1

HOO up-regulated

Gene Ontology category (level 3)	*Items*	*S*	*TS*	*Hyp*	*Genes*
response to external stimulus	GO:0009605	8(53)	912(22938)	1, 10E-03	Tpm1, Sdc1, Cyr61, Fn1, Ccnd1, Fgf10, Serpina3n, Timp1
behavior	GO:0007610	5(53)	443(22938)	3, 52E-03	Oxtr, Sncg, Penk1, Cyr61, Fgf10
cell adhesion	GO:0007155	6(53)	667(22938)	4, 25E-03	Vcan, Spon1, Tpm1, Sdc1, Cyr61, Fn1
actin filament-based process	GO:0030029	3(53)	224(22938)	1, 50E-02	Dbn1, Cnn3, Tpm1
response to abiotic stimulus	GO:0009628	4(53)	490(22938)	2, 65E-02	LOC363060, Hspb6, Hmgn1, Ccnd1

51 days

HCO down-regulated

Gene Ontology category (level 3)	*Items*	*S*	*TS*	*Hyp*	*Genes*
alcohol metabolic process	GO:0006066	22(120)	545(22938)	8, 86E-10	Mif, Lss, Adipoq, Bche, Taldo1, Pfkfb3, Dgat2, Pygl, Tkt, Insig1, Pdk1, Aldoa, Pc, Gpd1, Cat, LOC678700, G6pd, Mdh1, Pdhb, Sod1, Dhcr7, Cyb5r3
oxidation reduction	GO:0055114	22(120)	561(22938)	1, 59E-08	Cebpa, Gpd1, Sc5dl, Cat, LOC678700, G6pd, Retsat, Mdh1, Uqcrfs1, Aldh2, Hsd17b12, Pdhb, Sod1, Dhcr7, Cyb5r3, Sod3, Akr7a2, Prdx6, Akr1c14, Scd4, Scd1, Scd
response to biotic stimulus	GO:0009607	8(120)	289(22938)	1, 43E-04	Mx2, Mx1, Mgst1, Hsp90ab1, Ifitm2, Mif, Aldoa, Cebpa
multicellular organism growth	GO:0035264	4(120)	88(22938)	1, 18E-03	Gpam, G6pd, Sod1, Dhcr7
cytokine production	GO:0001816	5(120)	166(22938)	1, 82E-03	Fabp4, Adipoq, Cebpa, G6pd, Sod1
response to external stimulus	GO:0009605	12(120)	911(22938)	2, 97E-03	Acsl1, Cox4i1, Aacs, Ddt, Fabp4, Adora1, Mif, Adipoq, Bche, Cebpa, G6pd, Sod1
cellular response to stimulus	GO:0051716	8(120)	615(22938)	1, 56E-02	Lpin1, Mif, Insig1, Cebpa, Cat, Aldh2, Sod1, Prdx6
aging	GO:0007568	3(120)	125(22938)	2, 80E-02	Atp5g3, Mif, Sod1
cell death	GO:0008219	9(120)	827(22938)	2, 97E-02	Vdac1, Cstb, Dnase2a, Adora1, Adipoq, Cebpa, Cat, G6pd, Sod1

HOO down-regulated

Gene Ontology category (level 3)	*Items*	*S*	*TS*	*Hyp*	*Genes*
alcohol metabolic process	GO:0006066	23(98)	546(22938)	8, 13E-12	Lss, Hk2, Taldo1, Aldoa, Insig1, Bche, Tkt, Dgat2, Pygl, Pdk1, Pc, Mdh1, Nsdhl, Hsd11b1, Cyb5r3, Sod1, Ldhb, pdha1, Dhcr7, Gpd1, LOC678700, G6pd, Pdhb
oxidation reduction	GO:0055114	21(98)	562(22938)	2, 26E-09	Sod3, Mdh1, Nsdhl, Hsd11b1, Scd4, Scd1, Scd, Akr7a2, Cyb5r3, Acadsb, Sod1, Ldhb, pdha1, Hsd17b12, Dhcr7, Gpd1, LOC678700, G6pd, Aox4, Aox3, Pdhb
response to external stimulus	GO:0009605	13(98)	914(22938)	1, 37E-04	Gas6, Aacs, Pparg, Scnn1b, Fabp4, Acsl1, Timp3, Adora1, C6, tgfb3, Bche, Sod1, G6pd
multicellular organism growth	GO:0035264	4(98)	88(22938)	5, 55E-04	Gpam, Sod1, Dhcr7, G6pd
cytokine production	GO:0001816	4(98)	166(22938)	5, 64E-03	Pparg, Fabp4, Sod1, G6pd
aging	GO:0007568	3(98)	125(22938)	1, 65E-02	Timp3, tgfb3, Sod1
cell proliferation	GO:0008283	7(98)	747(22938)	4, 10E-02	S100a6, Pparg, Fabp4, Kit, Adora1, tgfb3, Dhcr7

100 days

LF-HOO down-regulated

Gene Ontology category (level 3)	*Items*	*S*	*TS*	*Hyp*	*Genes*
response to external stimulus	GO:0009605	5(34)	915(22938)	1, 06E-02	Aacs, Acsl1, Asns, Mif, Alb
cellular response to stimulus	GO:0051716	4(34)	616(22938)	1, 26E-02	Insig1, Asns, Mif, Alb
alcohol metabolic process	GO:0006066	3(34)	546(22938)	4, 65E-02	Pc, Insig1, Mif

246 days

HCO down-regulated

Gene Ontology category (level 3)	*Items*	*S*	*TS*	*Hyp*	*Genes*
alcohol metabolic process	GO:0006066	18(135)	547(22938)	3, 78E-05	Tkt, Dgat2, Taldo1, Pdk1, Chpt1, Bche, Pygl, Pc, Aldoa, Cyb5r3, Nsdhl, Pdhb, RGD1562690, Ldha, Mdh1, Dhcr7, Ldhb, Insig1
response to external stimulus	GO:0009605	15(135)	916(22938)	3, 31E-04	RT1-CE16, Gas6, Cmklr1, tgfb3, Cfb, Aacs, Scnn1b, C6, Cox4i1, Ddt, Bche, RGD1562690, Ldha, Pparg, Asns
oxidation reduction	GO:0055114	16(135)	563(22938)	2, 19E-02	Cdo1, Hsd17b12, Cyb5r3, Aox3, Nsdhl, Aldh6a1, Me1, Pdhb, RGD1562690, Ldha, Mdh1, Dhcr7, Ivd, Ldhb, Uqcrfs1, Akr7a2
cell death	GO:0008219	10(135)	828(22938)	2, 46E-02	Serpinb9, Sycp3, Vdac1, Cdkn2c, tgfb3, C6, Hspb1, RGD1562690, Ldha, Asns

LF-HCO down-regulated

Gene Ontology category (level 3)	*Items*	*S*	*TS*	*Hyp*	*Genes*
oxidation reduction	GO:0055114	20(94)	564(22938)	1, 16E-08	Uqcrfs1, Scd, Prdx6, Pdhb, Nsdhl, Me1, Mdh1, Ldhb, Hsd17b12, Gpd1, Etfb, Cyb5r3, Cdo1, Aox3, Aoc3, Aldh6a1, Akr7a2, Dhcr7, Ldha, G6pd
alcohol metabolic process	GO:0006066	17(94)	545(22938)	7, 43E-06	Pygl, Pdk1, Pc, Insig1, Dgat2, Chpt1, Aldoa, Pdhb, Nsdhl, Mdh1, Ldhb, Gpd1, Cyb5r3, Dhcr7, Ldha, G6pd, Bche
multicellular organism growth	GO:0035264	4(94)	86(22938)	4, 34E-04	Gpam, Tshr, Dhcr7, G6pd
cell death	GO:0008219	11(94)	825(22938)	5, 55E-04	Vdac1, Serpinb9, Ptma, Hspb1, Cidec, Cdkn2c, Ldha, G6pd, C6, Adora1, Tgfb3
cell proliferation	GO:0008283	7(94)	745(22938)	3, 34E-02	S100a6, Dhcr7, Ptma, Cdkn2c, Pparg, Adora1, Tgfb3

HOO down-regulated

Gene Ontology category (level 3)	*Items*	*S*	*TS*	*Hyp*	*Genes*
multicellular organism growth	GO:0035264	3(43)	88(22938)	6, 03E-04	Gpam, Dhcr7, G6pd
oxidation reduction	GO:0055114	5(43)	562(22938)	3, 87E-03	Dhcr7, Nsdhl, LOC678700, G6pd, pdha1
alcohol metabolic process	GO:0006066	10(43)	545(22938)	5, 00E-03	Chpt1, Pc, Aldoa, Pygl, Pdk1, Dhcr7, Nsdhl, LOC678700, G6pd, pdha1
response to external stimulus	GO:0009605	5(43)	915(22938)	2, 75E-02	Aacs, C6, Scnn1b, Cox4i1, G6pd

LF-HOO down-regulated

Gene Ontology category (level 3)	*Items*	*S*	*TS*	*Hyp*	*Genes*
alcohol metabolic process	GO:0006066	18(78)	545(22938)	2, 50E-08	Dgat2, Tkt, Prkaa2, Lss, Bche, Taldo1, Chpt1, Pdk1, Pygl, Aldoa, Pc, LOC678700, G6pd, Ldhb, Nsdhl, Mdh1, Dhcr7, Pdhb
multicellular organism growth	GO:0035264	4(78)	88(22938)	2, 32E-04	Tshr, Gpam, G6pd, Dhcr7
response to external stimulus	GO:0009605	9(78)	914(22938)	3, 79E-03	Acsl1, Asns, Cox4i1, C6, Scnn1g, Scnn1b, tgfb3, Bche, G6pd
oxidation reduction	GO:0055114	13(78)	561(22938)	5, 03E-03	Scd, Prdx6, LOC678700, G6pd, Aldh6a1, Ldhb, Nsdhl, Aox3, Hsd17b12, Mdh1, Akr7a2, Dhcr7, Pdhb
cell death	GO:0008219	8(78)	826(22938)	6, 95E-03	Cdkn2c, Vdac1, Ptma, Sycp3, Asns, C6, tgfb3, G6pd

[a]*: enriched Gene Ontology caterogies*
[b]*: number of genes in the input list that have been matched in the enriched set and the total number of genes in the list*
[c]*: number of genes in the reference list that match with the enriched set and total number of genes in the reference list*
[d]*: pvalue calculated by the hypergeometric function*
[e]*: the genes of the input list that match with the enriched items*
