# Supplementary material for: Diets high in corn oil or extra-virgin olive oil differentially modify the gene expression profile of the mammary gland and influence experimental breast cancer susceptibility
Source: Eur J Nutr. 2015 Jun 20;55:1397–409. doi: 10.1007/s00394-015-0958-2 (PMC4875377; doi:10.1007/s00394-015-0958-2)
Supplement: Supplementary file 3 — Enriched Gene Ontology (GO) categories in commonly or specifically modulated genes in mammary gland by effect of the high-fat diets at 36 days. Down- and up-modulated genes in each group were sub-classified in “common” (modulated in more than one group) or “specific” (found as modulated in one specific group) (PDF 64 kb) [file 394_2015_958_MOESM3_ESM.pdf]

Article title: Diets high in corn oil or extra virgin olive oil differentially modify the gene expression profile of the mammary gland and influence experimental breast cancer susceptibility

Journal name: European Journal of Nutrition

Authors names: Raquel Moral[1], Raquel Escrich[1], Montserrat Solanas[1], Elena Vela[1], M. Carme Ruíz de Villa[2], Eduard Escrich[1].

Affiliations: [1]Department of Cell Biology, Physiology and Immunology, Physiology Unit, Medicine School, Universitat Autònoma de Barcelona, 08193 Bellaterra, Barcelona, Spain; [2]Department of Statistics, Universitat de Barcelona, 08028 Barcelona, Spain.

Corresponding author: Dr. Eduard Escrich; Department of Cell Biology, Physiology and Immunology; Physiology Unit, Medicine School; Universitat Autònoma de Barcelona; 08193 Bellaterra (Barcelona), Spain. Phone: +34 93 581 14 23, Fax: +34 93 581 29 86. Eduard.Escrich@uab.es

Electronic Supplementary Material 3:

Supplementary Table 3

Enriched Gene Ontology (GO) categories in down- and up-modulated genes in mammary gland by effect of the experimental high fat diets.

Modulated genes were sub-classified in “common” (modulated in more than one group) or “specific” (found as modulated in one specific group)

Lists of “common” and “specific” genes found in high fat diet groups at 36 days of age were analyzed using Genecodis program by Babelomics platform setting GO annotations at level 3-6. Only statistically significant enriched categories are indicated.

36 days

Common down-regulated genes (in HCO and HOO)

(No significant enriched categories)

Specific down-regulated genes (only in HCO)

Gene Ontology category (levels 3-6)	*Items*[a]	*S*[b]	*TS*[c]	*Hyp*[d]	*Genes*[e]
immune response	GO:0006955	18(101)	560(22938)	4, 13E-06	RT1-Bb, Irf8, Il2rg, Hla-dmb, Cd79b, Cd37, Cd19, Ccl21b, RT1-Ba, Hla-dma, Rbm4b, Arbp, Cd74, Rab27a, Cd8a, Lyn, Cd28, Tgfb1
immune system development	GO:0002520	14(101)	304(22938)	6, 98E-06	Cd3d, LOC364236, Dnase2a, Satb1, Irf8, Il2rg, RT1-Ba, Hla-dma, Coro1a, Cd8a, Nme2, Lyn, Cd28, Tgfb1
tissue remodeling	GO:0002684	12(101)	197(22938)	7, 03E-06	Il2rg, Cd79b, Cd37, Cd19, RT1-Ba, Hla-dma, Coro1a, Cd5, Cd38, Lyn, Cd28, Tgfb1
T cell activation	GO:0042110	11(101)	172(22938)	2, 71E-05	Cd3d, Satb1, Il2rg, RT1-Ba, Hla-dma, Rab27a, Coro1a, Cd8a, Cd5, Cd28, Tgfb1
lymphocyte activation	GO:0046649	12(101)	237(22938)	5, 93E-05	Cd3d, Satb1, Il2rg, RT1-Ba, Hla-dma, Rab27a, Coro1a, Cd8a, Cd5, Cd38, Cd28, Tgfb1
cell proliferation	GO:0008283	12(101)	746(22938)	1, 08E-04	S100a6, Satb1, Rac2, Coro1a, Cd5, Id3, Cd38, Ptk2b, Npm1, Lyn, Cd28, Tgfb1
regulation of immune system process	GO:0002682	13(101)	311(22938)	1, 14E-04	Il2rg, Cd79b, Cd37, Cd19, RT1-Ba, Hla-dma, Coro1a, Cd5, Cd38, Nme2, Lyn, Cd28, Tgfb1
positive regulation of cell proliferation	GO:0008284	8(101)	337(22938)	1, 23E-04	Rac2, Coro1a, Id3, Cd38, Ptk2b, Lyn, Cd28, Tgfb1
cell activation	GO:0001775	13(101)	313(22938)	1, 23E-04	Cd3d, Satb1, Il2rg, RT1-Ba, Hla-dma, Rab27a, Coro1a, Cd8a, Cd5, Cd38, Lyn, Cd28, Tgfb1
homeostasis of number of cells	GO:0048872	5(101)	111(22938)	1, 33E-04	LOC364236, Dnase2a, Coro1a, Lyn, Tgfb1
response to organic cyclic substance	GO:0014070	6(101)	179(22938)	1, 43E-04	Adrbk1, Nedd8, Ptk2b, Lyn, Lef1, Tgfb1
response to wounding	GO:0009611	10(101)	546(22938)	1, 49E-04	Ptpn6, Ninj1, Rbm4b, Arbp, Rab27a, Cd8a, Id3, Lyn, Cd28, Tgfb1
regulation of cell communication	GO:0010646	13(101)	904(22938)	1, 68E-04	Rhoh, Rasgrp1, Dgkz, Cd8a, Cd5, Arrb2, Arhgef1, Cd38, Adrbk1, Ptk2b, Lyn, Lef1, Tgfb1
regulation of cell proliferation	GO:0042127	10(101)	584(22938)	2, 55E-04	Rac2, Coro1a, Cd5, Id3, Cd38, Ptk2b, Npm1, Lyn, Cd28, Tgfb1
cell death	GO:0008219	12(101)	825(22938)	2, 74E-04	Tp53inp1, Diablo, Dnase1l3, Dnase2a, Lyz2, Rab27a, Cd5, Id3, Ptk2b, Npm1, Cd28, Tgfb1
positive regulation of cell death	GO:0010942	7(101)	294(22938)	3, 26E-04	Tp53inp1, Diablo, Rab27a, Cd5, Id3, Cd28, Tgfb1
response to protein stimulus	GO:0051789	4(101)	76(22938)	3, 56E-04	Hsp90ab1, Id3, Lyn, Tgfb1
antigen processing and presentation of peptide antigen	GO:0048002	3(101)	33(22938)	4, 11E-04	RT1-Bb, RT1-Ba, Hla-dma

response to organic substance	GO:0010033	11(101)	746(22938)	4, 45E-04	Uqcrfs1, Hsp90ab1, Coro1a, Id3, Cd38, Adrbk1, Nedd8, Ptk2b, Lyn, Lef1, Tgfb1
bone remodeling	GO:0046849	3(101)	36(22938)	5, 32E-04	Rac2, Cd38, Tgfb1
B cell activation	GO:0042113	4(101)	89(22938)	6, 49E-04	Il2rg, Cd38, Cd28, Tgfb1
defense response	GO:0006952	9(101)	546(22938)	6, 89E-04	Lyz2, RT1-Ba, Rbm4b, Arbp, Rab27a, Cd8a, Lyn, Cd28, Tgfb1
apoptosis	GO:0006915	11(101)	789(22938)	7, 09E-04	Tp53inp1, Diablo, Dnase1l3, Dnase2a, Rab27a, Cd5, Id3, Ptk2b, Npm1, Cd28, Tgfb1
programmed cell death	GO:0012501	11(101)	797(22938)	7, 70E-04	Tp53inp1, Diablo, Dnase1l3, Dnase2a, Rab27a, Cd5, Id3, Ptk2b, Npm1, Cd28, Tgfb1
positive regulation of catalytic activity	GO:0043085	7(101)	341(22938)	7, 86E-04	Dgka, Gadd45b, Diablo, Dgkz, Ptk2b, Npm1, Tgfb1
positive regulation of cell activation	GO:0050867	8(101)	98(22938)	1, 21E-03	Il2rg, RT1-Ba, Hla-dma, Coro1a, Cd5, Cd38, Cd28, Tgfb1
protein complex assembly	GO:0006461	8(101)	477(22938)	1, 23E-03	Tuba1b, MGC72973, Vamp8, Hla-dma, Coro1a, Ptk2b, Npm1, Tgfb1
immunoglobulin production	GO:0002377	3(101)	48(22938)	1, 24E-03	Cd37, Cd28, Tgfb1
positive regulation of transferase activity	GO:0051347	5(101)	181(22938)	1, 24E-03	Dgka, Gadd45b, Dgkz, Ptk2b, Tgfb1
membrane organization	GO:0016044	6(101)	283(22938)	1, 59E-03	Vamp8, Cdc42se1, Timm13, Coro1a, Arrb2, Tgfb1
inflammatory response	GO:0006954	6(101)	296(22938)	2, 00E-03	Rbm4b, Arbp, Cd8a, Lyn, Cd28, Tgfb1
regulation of programmed cell death	GO:0043067	9(101)	640(22938)	2, 07E-03	Tp53inp1, Diablo, Rab27a, Cd5, Id3, Ptk2b, Npm1, Cd28, Tgfb1
erythrocyte homeostasis	GO:0034101	3(101)	58(22938)	2, 14E-03	LOC364236, Dnase2a, Lyn
inflammatory response to antigenic stimulus	GO:0002437	3(101)	59(22938)	2, 25E-03	Rbm4b, Arbp, Cd28
positive regulation of cell differentiation	GO:0045597	5(101)	212(22938)	2, 48E-03	Il2rg, RT1-Ba, Hla-dma, Lyn, Tgfb1
tissue homeostasis	GO:0001894	3(101)	62(22938)	2, 60E-03	Rac2, Coro1a, Cd38
mitochondrial transport	GO:0006839	3(101)	63(22938)	2, 72E-03	Timm13, Atp5o, Ucp2
cell cycle	GO:0007049	8(101)	543(22938)	2, 76E-03	Sept1, S100a6, Gadd45b, Tp53inp1, Id3, Npm1, Cd28, Tgfb1
regulation of cell differentiation	GO:0045595	7(101)	432(22938)	3, 02E-03	Il2rg, RT1-Ba, Hla-dma, Id3, Nme2, Lyn, Tgfb1
establishment of localization in cell	GO:0051649	11(101)	952(22938)	3, 13E-03	Timm13, Rac2, Cd74, Atp5o, Rab27a, Rhoh, Cd38, Ucp2, Adrbk1, Lyn, Tgfb1
response to carbohydrate stimulus	GO:0009743	3(101)	70(22938)	3, 66E-03	Ptk2b, Lyn, Tgfb1
homeostatic process	GO:0042592	10(101)	839(22938)	3, 89E-03	Prkcb, LOC364236, Dnase2a, Arbp, Rac2, Coro1a, Cd38, Ptk2b, Lyn, Tgfb1
leukocyte mediated immunity	GO:0002443	9(101)	162(22938)	4, 19E-03	RT1-Ba, Hla-dma, Rbm4b, Arbp, Rab27a, Cd8a, Lyn, Cd28, Tgfb1
tissue remodeling	GO:0048771	3(101)	74(22938)	4, 28E-03	Rac2, Cd38, Tgfb1

protein localization	GO:0008104	10(101)	869(22938)	4, 97E-03	Rab3ip, Hla-dma, Timm13, Rac2, Cd74, Rab27a, Rhoh, Nedd8, Lyn, Tgfb1
tissue homeostasis	GO:0000302	3(101)	79(22938)	5, 14E-03	Ucp2, Adrbk1, Ptk2b
vesicle-mediated transport	GO:0016192	7(101)	479(22938)	5, 29E-03	Vamp8, Cdc42se1, Rab27a, Coro1a, Arrb2, Lyn, Tgfb1
protein transport	GO:0015031	9(101)	752(22938)	5, 96E-03	Rab3ip, Hla-dma, Timm13, Rac2, Cd74, Rab27a, Rhoh, Lyn, Tgfb1
response to oxidative stress	GO:0006979	4(101)	165(22938)	6, 14E-03	Cd38, Ucp2, Adrbk1, Ptk2b
establishment of protein localization	GO:0045184	9(101)	762(22938)	6, 48E-03	Rab3ip, Hla-dma, Timm13, Rac2, Cd74, Rab27a, Rhoh, Lyn, Tgfb1
negative regulation of cell differentiation	GO:0051272	3(101)	89(22938)	7, 16E-03	Coro1a, Ptk2b, Tgfb1
endocytosis	GO:0006897	4(101)	174(22938)	7, 38E-03	Cdc42se1, Coro1a, Arrb2, Tgfb1
intracellular transport	GO:0046907	8(101)	657(22938)	8, 52E-03	Timm13, Rac2, Cd74, Atp5o, Rab27a, Rhoh, Ucp2, Tgfb1
cell adhesion	GO:0007155	8(101)	667(22938)	9, 28E-03	Gpnmb, Fnbp4, Selplg, Ninj1, Coro1a, Ptk2b, Lef1, Tgfb1
bone remodeling	GO:0006812	8(101)	668(22938)	9, 36E-03	Atp5i, Prkcb, Rac2, Atp5o, Slc38a1, Coro1a, Lyn, Tgfb1
cellular protein complex assembly	GO:0043623	4(101)	193(22938)	1, 05E-02	Tuba1b, Coro1a, Ptk2b, Tgfb1
heterocycle metabolic process	GO:0046483	6(101)	424(22938)	1, 12E-02	Phf11, Atp5i, Atp5o, Arrb2, Nme2, Tgfb1
regulation of catalytic activity	GO:0050790	7(101)	565(22938)	1, 25E-02	Dgka, Gadd45b, Diablo, Dgkz, Ptk2b, Npm1, Tgfb1
regulation of binding	GO:0051098	3(101)	114(22938)	1, 40E-02	Arrb2, Id3, Tgfb1
ion transport	GO:0006811	9(101)	874(22938)	1, 50E-02	Fxyd5, Atp5i, Prkcb, Rac2, Atp5o, Slc38a1, Coro1a, Lyn, Tgfb1
regulation of cell cycle	GO:0051726	4(101)	219(22938)	1, 61E-02	Gadd45b, Id3, Cd28, Tgfb1
DNA metabolic process	GO:0006259	6(101)	461(22938)	1, 63E-02	Dnase1l3, Dnase2a, Id3, Npm1, Cd28, Tgfb1
aging	GO:0007568	3(101)	124(22938)	1, 75E-02	Adrbk1, Npm1, Tgfb1
secretion by cell	GO:0032940	5(101)	341(22938)	1, 75E-02	Rab27a, Cd38, Adrbk1, Lyn, Tgfb1
nuclear transport	GO:0051169	4(101)	242(22938)	2, 23E-02	Rac2, Rab27a, Rhoh, Tgfb1
acute inflammatory response	GO:0002526	3(101)	138(22938)	2, 31E-02	Rbm4b, Arbp, Lyn
response to hypoxia	GO:0006935	3(101)	141(22938)	2, 45E-02	Rac2, Coro1a, Tgfb1
antigen processing and presentation of peptide or polysaccharide antigen via MHC class II	GO:0002504	4(101)	13(22938)	2, 46E-02	RT1-Bb, Hla-dmb, RT1-Ba, Hla-dma
cellular chemical homeostasis	GO:0055082	5(101)	373(22938)	2, 47E-02	Prkcb, Cd38, Ptk2b, Lyn, Tgfb1

cellular protein localization	GO:0034613	6(101)	509(22938)	2, 51E-02	Timm13, Rac2, Cd74, Rab27a, Rhoh, Tgfb1
cellular homeostasis	GO:0030334	3(101)	143(22938)	2, 54E-02	Coro1a, Ptk2b, Tgfb1
amine transport	GO:0015837	3(101)	144(22938)	2, 58E-02	Slc38a1, Adrbk1, Lyn
secretion	GO:0046903	5(101)	387(22938)	2, 83E-02	Rab27a, Cd38, Adrbk1, Lyn, Tgfb1
cytoskeleton organization	GO:0007010	5(101)	394(22938)	3, 03E-02	Tuba1b, Rac2, Coro1a, Ptk2b, Npm1
response to hormone stimulus	GO:0009725	5(101)	400(22938)	3, 20E-02	Uqcrfs1, Cd38, Ptk2b, Lyn, Tgfb1
ion homeostasis	GO:0050801	5(101)	406(22938)	3, 38E-02	Prkcb, Cd38, Ptk2b, Lyn, Tgfb1
response to hypoxia	GO:0001666	3(101)	163(22938)	3, 54E-02	Cd38, Ptk2b, Tgfb1
cellular chemical homeostasis	GO:0051270	3(101)	163(22938)	3, 54E-02	Coro1a, Ptk2b, Tgfb1
transmembrane transport	GO:0055085	3(101)	165(22938)	3, 65E-02	Atp5i, Timm13, Atp5o
cytokine production	GO:0001816	3(101)	165(22938)	3, 65E-02	Lyn, Cd28, Tgfb1
ion homeostasis	GO:0040012	3(101)	166(22938)	3, 71E-02	Coro1a, Ptk2b, Tgfb1
dephosphorylation	GO:0016311	3(101)	166(22938)	3, 71E-02	Dusp2, Ptpn6, Tgfb1
response to biotic stimulus	GO:0009607	4(101)	289(22938)	3, 90E-02	Mx1, Lyz2, Hsp90ab1, Tgfb1
protein complex assembly	GO:0008361	3(101)	176(22938)	4, 29E-02	Cd38, Ptk2b, Tgfb1
negative regulation of cell differentiation	GO:0045596	3(101)	180(22938)	4, 53E-02	Id3, Nme2, Tgfb1
response to drug	GO:0042493	4(101)	304(22938)	4, 55E-02	Uqcrfs1, Ptk2b, Lyn, Tgfb1
positive regulation of metabolic process	GO:0009893	7(101)	748(22938)	4, 72E-02	Ptk2b, Npm1, Nme2, Lyn, Lef1, Cd28, Tgfb1
cellular homeostasis	GO:0019725	5(101)	450(22938)	4, 88E-02	Prkcb, Cd38, Ptk2b, Lyn, Tgfb1

Specific up-regulated genes (only in HCO)

Gene Ontology category (levels 3-6)	*Items*	*S*	*TS*	*Hyp*	*Genes*
regulation of developmental process	GO:0050793	5(16)	819(22938)	1, 80E-04	Rtn4r, Itga7, Lep, Igf1, Alb
neurogenesis	GO:0022008	4(16)	573(22938)	5, 52E-04	Chrdl1, Rtn4r, Lep, Igf1
regulation of growth	GO:0040008	3(16)	251(22938)	6, 52E-04	Tshr, Lep, Igf1
regulation of cellular component organization	GO:0051128	3(16)	343(22938)	1, 61E-03	Rtn4r, Itga7, Igf1

cell development	GO:0048468	4(16)	844(22938)	2, 32E-03	Chrdl1, Rtn4r, Lep, Igf1
nervous system development	GO:0007399	4(16)	887(22938)	2, 79E-03	Chrdl1, Rtn4r, Lep, Igf1
regulation of cell differentiation	GO:0045595	3(16)	433(22938)	3, 12E-03	Rtn4r, Lep, Igf1
response to external stimulus	GO:0009605	4(16)	915(22938)	3, 12E-03	F13a1, Lep, Igf1, Alb
response to organic substance	GO:0010033	3(16)	748(22938)	1, 41E-02	Lep, Igf1, Alb
regulation of cell communication	GO:0010646	3(16)	906(22938)	2, 34E-02	S100b, Lep, Igf1

Specific down-regulated genes (only in HOO)

Gene Ontology category (levels 3-6)	*Items*	*S*	*TS*	*Hyp*	*Genes*
alcohol metabolic process	GO:0006066	14(43)	546(22938)	6, 61E-08	Pfkfb3, Pdk1, Tkt, Taldo1, Pygl, Bche, Insig1, Dhcr7, Cyb5r3, Chpt1, Pdhb, Pdha1, Pc, Pfkl
cellular carbohydrate metabolic process	GO:0044262	10(43)	341(22938)	5, 74E-05	Pfkfb3, Lalba, Acly, Tkt, Taldo1, Pygl, Pdhb, Pdha1, Pc, Pfkl
carbohydrate biosynthetic process	GO:0016051	4(43)	141(22938)	1, 40E-04	Lalba, Tkt, Pygl, Pc
steroid metabolic process	GO:0008202	4(43)	177(22938)	3, 34E-04	Insig1, Hsd17b12, Dhcr7, Cyb5r3
lipid biosynthetic process	GO:0008610	9(43)	299(22938)	3, 68E-04	Hsd17b12, Dhcr7, Cyb5r3, Chpt1, Scd, Pc, Gpam, Elovl6, Acaca
generation of precursor metabolites and energy	GO:0006091	5(43)	335(22938)	3, 94E-04	Pfkfb3, Pygl, Pdhb, Pdha1, Pfkl
response to organic substance	GO:0010033	7(43)	746(22938)	4, 34E-04	Tgfb3, Tf, Bche, Insig1, Me1, Acaca, Pfkl
lipid metabolic process	GO:0006629	12(43)	735(22938)	6, 60E-04	Insig1, Hsd17b12, Dhcr7, Cyb5r3, Chpt1, Slc27a1, Scd, Pc, Gpam, Elovl6, Acadsb, Acaca
cellular lipid metabolic process	GO:0044255	11(43)	622(22938)	1, 40E-03	Insig1, Hsd17b12, Dhcr7, Cyb5r3, Chpt1, Slc27a1, Scd, Gpam, Elovl6, Acadsb, Acaca
chemical homeostasis	GO:0048878	5(43)	483(22938)	2, 02E-03	Pygl, Slc2a4, Tf, Scd, Acaca
carbohydrate metabolic process	GO:0005975	11(43)	674(22938)	3, 18E-03	Pfkfb3, Pdk1, Lalba, Acly, Tkt, Taldo1, Pygl, Pdhb, Pdha1, Pc, Pfkl
hexose metabolic process	GO:0019318	8(43)	291(22938)	6, 01E-03	Pfkfb3, Pdk1, Tkt, Taldo1, Pdhb, Pdha1, Pc, Pfkl
response to hormone stimulus	GO:0009725	4(43)	399(22938)	6, 52E-03	Tgfb3, Bche, Insig1, Me1
carboxylic acid metabolic process	GO:0019752	10(43)	566(22938)	7, 12E-03	Slc27a1, Scd, Pdhb, Pdha1, Pc, Me1, Gpam, Elovl6, Acadsb, Acaca
organic acid metabolic process	GO:0006082	10(43)	567(22938)	7, 24E-03	Slc27a1, Scd, Pdhb, Pdha1, Pc, Me1, Gpam, Elovl6, Acadsb, Acaca
response to steroid hormone stimulus	GO:0048545	3(43)	236(22938)	9, 80E-03	Tgfb3, Bche, Insig1
homeostatic process	GO:0042592	5(43)	840(22938)	1, 98E-02	Pygl, Slc2a4, Tf, Scd, Acaca

transmission of nerve impulse	GO:0019226	3(43)	355(22938)	2, 88E-02	Tf, Bche, Scd

Specific up-regulated genes (only in HOO)

Gene Ontology category (levels 3-6)	*Items*	*S*	*TS*	*Hyp*	*Genes*
muscle contraction	GO:0006936	7(44)	119(22938)	2, 77E-04	Tnni2, Myl1, Actn3, Pgam2, Hspb6, Mylpf, Tpm1
response to external stimulus	GO:0009605	8(44)	913(22938)	3, 03E-04	Timp1, Fn1, Fgf10, Eno3, Ccnd1, Cyr61, Sdc1, Tpm1
response to organic substance	GO:0010033	7(44)	747(22938)	5, 06E-04	Atp5g2, Socs2, Pck1, Timp1, Ccnd1, Cyr61, Sdc1
response to wounding	GO:0009611	6(44)	546(22938)	5, 79E-04	Timp1, Fn1, Fgf10, Eno3, Sdc1, Tpm1
response to hormone stimulus	GO:0009725	5(44)	399(22938)	9, 66E-04	Socs2, Pck1, Timp1, Ccnd1, Sdc1
cell adhesion	GO:0007155	6(44)	667(22938)	1, 63E-03	Vcan, Spon1, Fn1, Cyr61, Sdc1, Tpm1
aging	GO:0007568	3(44)	125(22938)	1, 78E-03	Socs2, Timp1, Eno3
hexose metabolic process	GO:0019318	4(44)	291(22938)	2, 31E-03	Pfkm, Pgam2, Pck1, Eno3
response to metal ion	GO:0010038	3(44)	145(22938)	2, 71E-03	Pgam2, Ccnd1, Sdc1
regulation of locomotion	GO:0040012	3(44)	166(22938)	3, 97E-03	Fgf10, Cyr61, Tpm1
cellular carbohydrate metabolic process	GO:0044262	4(44)	341(22938)	4, 08E-03	Pfkm, Pgam2, Pck1, Eno3
response to inorganic substance	GO:0010035	3(44)	170(22938)	4, 24E-03	Pgam2, Ccnd1, Sdc1
response to peptide hormone stimulus	GO:0043434	3(44)	188(22938)	5, 61E-03	Socs2, Pck1, Timp1
tissue development	GO:0009888	5(44)	606(22938)	5, 88E-03	Fgf10, Eno3, Ccnd1, Sdc1, Mylpf
organ morphogenesis	GO:0009887	5(44)	675(22938)	9, 15E-03	Hmgn1, Fgf10, Ccnd1, Cyr61, Sdc1
response to steroid hormone stimulus	GO:0048545	3(44)	236(22938)	1, 04E-02	Socs2, Ccnd1, Sdc1
cellular response to DNA damage stimulus	GO:0034984	3(44)	250(22938)	1, 22E-02	Pcna, Hmgn1, Ccnd1
response to abiotic stimulus	GO:0009628	4(44)	490(22938)	1, 42E-02	LOC363060, Hspb6, Hmgn1, Ccnd1
response to DNA damage stimulus	GO:0006974	3(44)	278(22938)	1, 62E-02	Pcna, Hmgn1, Ccnd1
regulation of developmental process	GO:0050793	5(44)	819(22938)	1, 97E-02	Timp1, Fn1, Fgf10, Ccnd1, Cyr61
alcohol metabolic process	GO:0006066	4(44)	546(22938)	2, 03E-02	Pfkm, Pgam2, Pck1, Eno3
cellular response to stimulus	GO:0051716	4(44)	615(22938)	2, 98E-02	Pcna, Socs2, Hmgn1, Ccnd1

cation transport	GO:0006812	4(44)	667(22938)	3, 85E-02	Tnni2, Atp5g2, Tpm1, Dbn1
carbohydrate metabolic process	GO:0005975	4(44)	674(22938)	3, 97E-02	Pfkm, Pgam2, Pck1, Eno3
cellular response to stress	GO:0033554	3(44)	429(22938)	4, 89E-02	Pcna, Hmgn1, Ccnd1

[a]: *enriched Gene Ontology caterogies*
[b]: *number of genes in the input list that have been matched in the enriched set and the total number of genes in the list*
[c]: *number of genes in the reference list that match with the enriched set and total number of genes in the reference list*
[d]: *pvalue calculated by the hypergeometric function*
[e]: *the genes of the input list that match with the enriched items*
