# Supplementary material for: Diets high in corn oil or extra-virgin olive oil differentially modify the gene expression profile of the mammary gland and influence experimental breast cancer susceptibility
Source: Eur J Nutr. 2015 Jun 20;55:1397–409. doi: 10.1007/s00394-015-0958-2 (PMC4875377; doi:10.1007/s00394-015-0958-2)
Supplement: Supplementary file 4 — Enriched Gene Ontology (GO) categories in commonly or specifically modulated genes in mammary gland by effect of the high-fat diets at 51 days. Down- and up-modulated genes in each group were sub-classified in “common” (modulated in more than one group) or “specific” (found as modulated in one specific group) (PDF 48 kb) [file 394_2015_958_MOESM4_ESM.pdf]

Article title: Diets high in corn oil or extra virgin olive oil differentially modify the gene expression profile of the mammary gland and influence experimental breast cancer susceptibility

Journal name: European Journal of Nutrition

Authors names: Raquel Moral[1], Raquel Escrich[1], Montserrat Solanas[1], Elena Vela[1], M. Carme Ruíz de Villa[2], Eduard Escrich[1].

Affiliations: [1]Department of Cell Biology, Physiology and Immunology, Physiology Unit, Medicine School, Universitat Autònoma de Barcelona, 08193 Bellaterra, Barcelona, Spain; [2]Department of Statistics, Universitat de Barcelona, 08028 Barcelona, Spain.

Corresponding author: Dr. Eduard Escrich; Department of Cell Biology, Physiology and Immunology; Physiology Unit, Medicine School; Universitat Autònoma de Barcelona; 08193 Bellaterra (Barcelona), Spain. Phone: +34 93 581 14 23, Fax: +34 93 581 29 86. Eduard.Escrich@uab.es

Electronic Supplementary Material 4:

Supplementary Table 4

Enriched Gene Ontology (GO) categories in down- and up-modulated genes in mammary gland by effect of the experimental high fat diets.

Modulated genes were sub-classified in “common” (modulated in more than one group) or “specific” (found as modulated in one specific group)

Lists of <u>“common” and “specific” genes found in high fat diet groups at 51 days of age</u> were analyzed using Genecodis program by Babelomics platform setting GO annotations at level 3-6. Only statistically significant enriched categories are indicated.

51 days

Common down-regulated genes (in HCO and HOO)

Gene Ontology category (levels 3-6)	*Items[a]*	*S[b]*	*TS[c]*	*Hyp[d]*	*Genes[e]*
alcohol metabolic process	GO:0006066	18(61)	546(22938)	2, 32E-15	Pdk1, Tkt, Taldo1, Pygl, Aldoa, G6pd, Bche, Sod1, Lss, Insig1, Dhcr7, Dgat2, Cyb5r3, Pdhb, Pc, Mdh1, Ldhb, Gpd1
lipid metabolic process	GO:0006629	19(61)	735(22938)	2, 72E-09	Sod1, Lss, Lpin1, Insig1, Hsd17b12, Dhcr7, Dgat2, Cyb5r3, Akr1c14, Slc27a1, Scd, Pc, Gpd1, Gpam, Fabp4, Elovl6, Acsl1, Acaca, Adora1
cellular lipid metabolic process	GO:0044255	16(61)	622(22938)	4, 62E-07	Sod1, Lss, Insig1, Hsd17b12, Dhcr7, Dgat2, Cyb5r3, Akr1c14, Slc27a1, Scd, Gpd1, Gpam, Fabp4, Elovl6, Acsl1, Acaca
cellular carbohydrate metabolic process	GO:0044262	12(61)	341(22938)	8, 71E-06	Acly, Tkt, Taldo1, Pygl, Aldoa, G6pd, Dgat2, Pdhb, Pc, Mdh1, Ldhb, Gpd1
oxidation reduction	GO:0055114	14(61)	564(22938)	1, 96E-05	Sod3, Akr7a2, G6pd, Sod1, Hsd17b12, Dhcr7, Cyb5r3, Akr1c14, Scd, Pdhb, Me1, Mdh1, Ldhb, Gpd1
organic acid metabolic process	GO:0006082	13(61)	567(22938)	2, 49E-05	Slc27a1, Scd, Pdhb, Pc, Me1, Mdh1, Ldhb, Gpd1, Gpam, Fabp4, Elovl6, Acsl1, Acaca
lipid biosynthetic process	GO:0008610	11(61)	299(22938)	3, 60E-05	Sod1, Lss, Hsd17b12, Dhcr7, Dgat2, Cyb5r3, Scd, Pc, Gpam, Elovl6, Acaca
cofactor metabolic process	GO:0051186	10(61)	230(22938)	4, 87E-05	Tkt, Taldo1, Mgst1, G6pd, Sod1, Pdhb, Mdh1, Ldhb, Gpd1, Acaca
carbohydrate metabolic process	GO:0005975	13(61)	674(22938)	1, 95E-04	Pdk1, Acly, Tkt, Taldo1, Pygl, Aldoa, G6pd, Dgat2, Pdhb, Pc, Mdh1, Ldhb, Gpd1
carboxylic acid metabolic process	GO:0019752	13(61)	566(22938)	2, 44E-04	Slc27a1, Scd, Pdhb, Pc, Me1, Mdh1, Ldhb, Gpd1, Gpam, Fabp4, Elovl6, Acsl1, Acaca
generation of precursor metabolites and energy	GO:0006091	6(61)	335(22938)	2, 62E-04	Pygl, Atp5o, Aldoa, Pdhb, Mdh1, Ldhb
chemical homeostasis	GO:0048878	7(61)	483(22938)	2, 87E-04	Pygl, Slc2a4, Sod1, Scd, Fabp4, Acaca, Adora1
hexose metabolic process	GO:0019318	10(61)	291(22938)	4, 72E-04	Pdk1, Tkt, Taldo1, Aldoa, G6pd, Pdhb, Pc, Mdh1, Ldhb, Gpd1
fat cell differentiation	GO:0045444	3(61)	60(22938)	5, 50E-04	Slc2a4, Lpin1, Fabp4
regulation of multicellular organism growth	GO:0040014	3(61)	61(22938)	5, 77E-04	G6pd, Sod1, Gpam
mitochondrial transport	GO:0006839	3(61)	63(22938)	6, 34E-04	Slc25a10, Slc25a1, Atp5o
response to hormone stimulus	GO:0009725	6(61)	399(22938)	6, 61E-04	Aldoa, Bche, Lpin1, Insig1, Me1, Fabp4
regulation of blood vessel size	GO:0050880	3(61)	77(22938)	1, 14E-03	G6pd, Sod1, Adora1
response to organic cyclic substance	GO:0014070	4(61)	178(22938)	1, 30E-03	Aldoa, G6pd, Acsl1, Acaca

homeostatic process	GO:0042592	8(61)	840(22938)	1, 65E-03	Pygl, Slc2a4, G6pd, Sod1, Scd, Fabp4, Acaca, Adora1
regulation of lipid metabolic process	GO:0019216	3(61)	88(22938)	1, 67E-03	Sod1, Dhcr7, Adora1
peptide metabolic process	GO:0006518	3(61)	88(22938)	1, 67E-03	Mgst1, G6pd, Sod1
regulation of blood pressure	GO:0008217	3(61)	95(22938)	2, 08E-03	G6pd, Sod1, Adora1
sulfur metabolic process	GO:0006790	3(61)	98(22938)	2, 27E-03	Mgst1, G6pd, Sod1
response to nutrient levels	GO:0031667	4(61)	227(22938)	3, 14E-03	G6pd, Bche, Sod1, Acsl1
response to steroid hormone stimulus	GO:0048545	4(61)	236(22938)	3, 60E-03	Aldoa, Bche, Insig1, Fabp4
response to extracellular stimulus	GO:0009991	4(61)	245(22938)	4, 12E-03	G6pd, Bche, Sod1, Acsl1
regulation of growth	GO:0040008	4(61)	250(22938)	4, 42E-03	Tkt, G6pd, Sod1, Gpam
cellular macromolecule catabolic process	GO:0044265	11(61)	485(22938)	5, 44E-03	Ubc, Tkt, Taldo1, Pygl, Aldoa, G6pd, Sod1, Pdhb, Mdh1, Ldhb, Gpd1
glycerolipid metabolic process	GO:0046486	3(61)	136(22938)	5, 71E-03	Dgat2, Gpd1, Gpam
response to oxidative stress	GO:0006979	3(61)	165(22938)	9, 70E-03	Sod3, G6pd, Sod1
cytokine production	GO:0001816	3(61)	166(22938)	9, 86E-03	G6pd, Sod1, Fabp4
blood circulation	GO:0008015	3(61)	184(22938)	1, 30E-02	G6pd, Sod1, Adora1
transmission of nerve impulse	GO:0019226	4(61)	355(22938)	1, 47E-02	Bche, Sod1, Scd, Adora1
cellular amino acid and derivative metabolic process	GO:0006519	4(61)	357(22938)	1, 50E-02	Mgst1, G6pd, Bche, Sod1
vitamin metabolic process	GO:0006766	6(61)	99(22938)	2, 54E-02	Tkt, Taldo1, G6pd, Mdh1, Ldhb, Gpd1
heterocycle metabolic process	GO:0046483	4(61)	424(22938)	2, 63E-02	Tkt, Pygl, Atp5o, Adora1
response to external stimulus	GO:0009605	6(61)	913(22938)	3, 40E-02	G6pd, Bche, Sod1, Fabp4, Acsl1, Adora1
response to organic substance	GO:0010033	11(61)	746(22938)	3, 80E-02	Mgst1, Aldoa, G6pd, Bche, Sod1, Lpin1, Insig1, Me1, Fabp4, Acsl1, Acaca
response to biotic stimulus	GO:0009607	3(61)	289(22938)	4, 17E-02	Ifitm2, Mgst1, Aldoa
steroid metabolic process	GO:0008202	7(61)	177(22938)	4, 44E-02	Sod1, Lss, Insig1, Hsd17b12, Dhcr7, Cyb5r3, Akr1c14

Specific down-regulated genes (only in HCO)

Gene Ontology category (levels 3-6)	*Items*	*S*	*TS*	*Hyp*	*Genes*
lipid catabolic process	GO:0016042	4(38)	152(22938)	1, 15E-04	Smpdl3a, Prdx6, Echs1, Cebpa
transmembrane transport	GO:0055085	4(38)	165(22938)	1, 57E-04	Atp5i, Atp5e, Atp5g3, Atp5g2

response to organic substance	GO:0010033	7(38)	746(22938)	1, 96E-04	Hsp90ab1, Atp5g3, Atp5g2, Uqcrfs1, Aldh2, Mif, Cebpa
generation of precursor metabolites and energy	GO:0006091	9(38)	334(22938)	2, 97E-04	Atp5i, Atp5e, Atp5g3, Atp5g2, Uqcrfs1, Cat, Pfkfb3, Mif, Cebpa
ion transport	GO:0006811	7(38)	873(22938)	5, 08E-04	Slc36a2, Atp5i, Atp5e, Atp5g3, Atp5g2, Vdac1, Mif
response to biotic stimulus	GO:0009607	4(38)	289(22938)	1, 30E-03	Mx1, Hsp90ab1, Mif, Cebpa
response to nutrient	GO:0007584	3(38)	158(22938)	2, 27E-03	Cox4i1, Mif, Cebpa
cellular response to stimulus	GO:0051716	5(38)	616(22938)	3, 31E-03	Aldh2, Prdx6, Cat, Mif, Cebpa
response to hormone stimulus	GO:0009725	4(38)	398(22938)	4, 14E-03	Uqcrfs1, Aldh2, Mif, Cebpa
heterocycle metabolic process	GO:0046483	4(38)	424(22938)	5, 17E-03	Atp5i, Atp5e, Atp5g3, Atp5g2
response to other organism	GO:0051707	3(38)	225(22938)	6, 10E-03	Mx1, Mif, Cebpa
response to nutrient levels	GO:0031667	3(38)	227(22938)	6, 25E-03	Cox4i1, Mif, Cebpa
response to steroid hormone stimulus	GO:0048545	3(38)	235(22938)	6, 88E-03	Aldh2, Mif, Cebpa
response to extracellular stimulus	GO:0009991	3(38)	245(22938)	7, 71E-03	Cox4i1, Mif, Cebpa
cellular macromolecule catabolic process	GO:0044265	4(38)	485(22938)	8, 25E-03	Pfkfb3, Mif, Dnase2a, Cebpa
apoptosis	GO:0006915	5(38)	790(22938)	9, 37E-03	Vdac1, Cstb, Cat, Dnase2a, Cebpa
programmed cell death	GO:0012501	5(38)	798(22938)	9, 76E-03	Vdac1, Cstb, Cat, Dnase2a, Cebpa
cell death	GO:0008219	5(38)	826(22938)	1, 12E-02	Vdac1, Cstb, Cat, Dnase2a, Cebpa
inflammatory response	GO:0006954	3(38)	294(22938)	1, 26E-02	Ddt, Mif, Cebpa
immune system development	GO:0002520	3(38)	304(22938)	1, 38E-02	LOC364236, Dnase2a, Cebpa
cellular carbohydrate metabolic process	GO:0044262	3(38)	338(22938)	1, 83E-02	Pfkfb3, Mif, Cebpa
central nervous system development	GO:0007417	3(38)	374(22938)	2, 38E-02	Pfkfb3, Mif, Cebpa
cellular response to stress	GO:0033554	3(38)	430(22938)	3, 40E-02	Prdx6, Cat, Mif
behavior	GO:0007610	3(38)	443(22938)	3, 67E-02	Cstb, Mif, Cebpa
homeostatic process	GO:0042592	4(38)	839(22938)	4, 91E-02	Prdx6, LOC364236, Dnase2a, Cebpa

Specific down-regulated genes (only in HOO)

Gene Ontology category (levels 3-6)	*Items*	*S*	*TS*	*Hyp*	*Genes*
response to external stimulus	GO:0009605	6(23)	915(22938)	2, 23E-04	Scnn1b, Gas6, Tgfb3, C6, Timp3, Pparg

tissue development	GO:0009888	5(23)	607(22938)	2, 89E-04	Gas6, Tgfb3, Timp3, Nsdhl, Pparg
cellular carbohydrate metabolic process	GO:0044262	4(23)	340(22938)	3, 36E-04	Lalba, Hk2, Nsdhl, Hsd11b1
GO:0016052	GO:0016052	3(23)	143(22938)	3, 83E-04	Chi3l1, Hk2, Hsd11b1
carbohydrate metabolic process	GO:0005975	5(23)	673(22938)	4, 65E-04	Lalba, Chi3l1, Hk2, Nsdhl, Hsd11b1
reproductive process in a multicellular organism	GO:0048609	4(23)	405(22938)	6, 50E-04	Rgs2, Tgfb3, Hk2, Kit
response to steroid hormone stimulus	GO:0048545	3(23)	237(22938)	1, 66E-03	Tgfb3, Timp3, Pparg
nuclear transport	GO:0051169	3(23)	242(22938)	1, 76E-03	Rnd3, Rab34, Tgfb3
regulation of catalytic activity	GO:0050790	4(23)	567(22938)	2, 25E-03	Tgfb3, C6, Kit, Pparg
positive regulation of cell death	GO:0010942	3(23)	294(22938)	3, 05E-03	Tgfb3, C6, Timp3
cellular lipid metabolic process	GO:0044255	4(23)	622(22938)	3, 15E-03	Nsdhl, Kit, Hsd11b1, Pparg
regulation of programmed cell death	GO:0043067	4(23)	642(22938)	3, 52E-03	Tgfb3, C6, Timp3, Kit
positive regulation of catalytic activity	GO:0043085	3(23)	343(22938)	4, 70E-03	Tgfb3, C6, Kit
lipid metabolic process	GO:0006629	4(23)	736(22938)	5, 72E-03	Nsdhl, Kit, Hsd11b1, Pparg
protein transport	GO:0015031	4(23)	752(22938)	6, 17E-03	Rnd3, Rab34, Tgfb3, Pparg
establishment of protein localization	GO:0045184	4(23)	762(22938)	6, 47E-03	Rnd3, Rab34, Tgfb3, Pparg
response to hormone stimulus	GO:0009725	3(23)	400(22938)	7, 19E-03	Tgfb3, Timp3, Pparg
programmed cell death	GO:0012501	4(23)	799(22938)	7, 63E-03	Tgfb3, C6, Timp3, Kit
cell death	GO:0008219	4(23)	827(22938)	8, 60E-03	Tgfb3, C6, Timp3, Kit
regulation of cell differentiation	GO:0045595	3(23)	433(22938)	8, 94E-03	Tgfb3, Kit, Pparg
protein localization	GO:0008104	4(23)	869(22938)	1, 02E-02	Rnd3, Rab34, Tgfb3, Pparg
cellular macromolecule catabolic process	GO:0044265	3(23)	484(22938)	1, 21E-02	Timp3, Hk2, Hsd11b1
response to abiotic stimulus	GO:0009628	3(23)	491(22938)	1, 26E-02	Timp3, Kit, Pparg
cellular protein localization	GO:0034613	3(23)	509(22938)	1, 38E-02	Rnd3, Rab34, Tgfb3
alcohol metabolic process	GO:0006066	3(23)	545(22938)	1, 66E-02	Hk2, Nsdhl, Hsd11b1
negative regulation of metabolic process	GO:0009892	3(23)	547(22938)	1, 68E-02	Tgfb3, Timp3, Pparg
oxidation reduction	GO:0055114	3(23)	562(22938)	1, 80E-02	Aox3, Nsdhl, Hsd11b1

regulation of cell proliferation	GO:0042127	3(23)	584(22938)	1, 99E-02	Tgfb3, Kit, Pparg
intracellular transport	GO:0046907	3(23)	657(22938)	2, 71E-02	Rnd3, Rab34, Tgfb3
organ morphogenesis	GO:0009887	3(23)	675(22938)	2, 90E-02	Gas6, Tgfb3, Pparg
regulation of signal transduction	GO:0009966	3(23)	734(22938)	3, 59E-02	Rgs2, Tgfb3, Kit
cell proliferation	GO:0008283	3(23)	747(22938)	3, 75E-02	Tgfb3, Kit, Pparg
response to organic substance	GO:0010033	3(23)	748(22938)	3, 77E-02	Tgfb3, Timp3, Pparg
positive regulation of metabolic process	GO:0009893	3(23)	749(22938)	3, 78E-02	Tgfb3, Kit, Pparg
apoptosis	GO:0006915	3(23)	791(22938)	4, 33E-02	Tgfb3, C6, Timp3
regulation of developmental process	GO:0050793	3(23)	819(22938)	4, 72E-02	Tgfb3, Kit, Pparg

[a]*: enriched Gene Ontology caterogies*
[b]*: number of genes in the input list that have been matched in the enriched set and the total number of genes in the list*
[c]*: number of genes in the reference list that match with the enriched set and total number of genes in the reference list*
[d]*: pvalue calculated by the hypergeometric function*
[e]*: the genes of the input list that match with the enriched items*
