# Supplementary material for: Diets high in corn oil or extra-virgin olive oil differentially modify the gene expression profile of the mammary gland and influence experimental breast cancer susceptibility
Source: Eur J Nutr. 2015 Jun 20;55:1397–409. doi: 10.1007/s00394-015-0958-2 (PMC4875377; doi:10.1007/s00394-015-0958-2)
Supplement: Supplementary file 5 — Enriched Gene Ontology (GO) categories in commonly or specifically modulated genes in mammary gland by effect of the high-fat diets at 246 days. Down- and up-modulated genes in each group were sub-classified in “common” (modulated in more than one group) or “specific” (found as modulated in one specific group) (PDF 37 kb) [file 394_2015_958_MOESM5_ESM.pdf]

Article title: Diets high in corn oil or extra virgin olive oil differentially modify the gene expression profile of the mammary gland and influence experimental breast cancer susceptibility

Journal name: European Journal of Nutrition

Authors names: Raquel Moral[1], Raquel Escrich[1], Montserrat Solanas[1], Elena Vela[1], M. Carme Ruíz de Villa[2], Eduard Escrich[1].

Affiliations: [1]Department of Cell Biology, Physiology and Immunology, Physiology Unit, Medicine School, Universitat Autònoma de Barcelona, 08193 Bellaterra, Barcelona, Spain; [2]Department of Statistics, Universitat de Barcelona, 08028 Barcelona, Spain.

Corresponding author: Dr. Eduard Escrich; Department of Cell Biology, Physiology and Immunology; Physiology Unit, Medicine School; Universitat Autònoma de Barcelona; 08193 Bellaterra (Barcelona), Spain. Phone: +34 93 581 14 23, Fax: +34 93 581 29 86. Eduard.Escrich@uab.es

Electronic Supplementary Material 5:

Supplementary Table 5

Enriched Gene Ontology (GO) categories in down- and up-modulated genes in mammary gland by effect of the experimental high fat diets.

Modulated genes were sub-classified in "common" (modulated in more than one group) or "specific" (found as modulated in one specific group)

Lists of “specific” and “common” genes found in high fat diet groups at 246 days of age were analyzed using Genecodis program by Babelomics platform setting GO annotations at level 3-6. Only statistically significant enriched categories are indicated.

246 days

Common down-regulated genes (in HCO, LF-HCO, HOO and LF-HOO)

Gene Ontology category (levels 3-6)	*Items*[a]	*S*[b]	*TS*[c]	*Hyp*[d]	*Genes*[e]
hexose metabolic process	GO:0019318	4(21)	291(22938)	1,28E-04	Pdk1, Aldoa, Pdhb, Pc
carboxylic acid metabolic process	GO:0019752	5(21)	566(22938)	1,32E-04	Pdhb, Pc, Me1, Gpam, Elovl6
organic acid metabolic process	GO:0006082	5(21)	567(22938)	1,33E-04	Pdhb, Pc, Me1, Gpam, Elovl6
cellular lipid metabolic process	GO:0044255	5(21)	622(22938)	2,05E-04	Nsdhl, Dhcr7, Chpt1, Gpam, Elovl6
carbohydrate biosynthetic process	GO:0016051	3(21)	141(22938)	2,79E-04	Pygl, Nsdhl, Pc
alcohol metabolic process	GO:0006066	8(21)	546(22938)	1,52E-03	Pdk1, Pygl, Aldoa, Nsdhl, Dhcr7, Chpt1, Pdhb, Pc
oxidation reduction	GO:0055114	4(21)	564(22938)	1,55E-03	Nsdhl, Dhcr7, Pdhb, Me1
generation of precursor metabolites and energy	GO:0006091	3(21)	335(22938)	3,38E-03	Pygl, Aldoa, Pdhb
cellular macromolecule catabolic process	GO:0044265	3(21)	485(22938)	9,41E-03	Pygl, Aldoa, Pdhb
lipid biosynthetic process	GO:0008610	6(21)	298(22938)	2,11E-02	Nsdhl, Dhcr7, Chpt1, Pc, Gpam, Elovl6
cellular carbohydrate metabolic process	GO:0044262	6(21)	341(22938)	4,64E-02	Acly, Pygl, Aldoa, Nsdhl, Pdhb, Pc
response to external stimulus	GO:0009605	3(21)	915(22938)	4,93E-02	Cox4i1, C6, Scnn1b

Common down-regulated genes (in HCO, LF-HCO and LF-HOO)

Gene Ontology category (levels 3-6)	*Items*	*S*	*TS*	*Hyp*	*Genes*
oxidation reduction	GO:0055114	5(21)	562(22938)	1,27E-04	Prdx6, Mdh1, Ldhb, Hsd17b12, Aox3
alcohol metabolic process	GO:0006066	4(21)	546(22938)	1,38E-03	Dgat2, Bche, Mdh1, Ldhb
cellular lipid metabolic process	GO:0044255	4(21)	623(22938)	2,23E-03	Dgat2, Echs1, Prdx6, Hsd17b12
cellular carbohydrate metabolic process	GO:0044262	3(21)	341(22938)	3,55E-03	Dgat2, Mdh1, Ldhb
lipid metabolic process	GO:0006629	4(21)	737(22938)	4,09E-03	Dgat2,Echs1,Prdx6,Hsd17b12
carboxylic acid metabolic process	GO:0019752	3(21)	566(22938)	1,43E-02	Echs1,Mdh1,Ldhb
organic acid metabolic process	GO:0006082	3(21)	567(22938)	1,43E-02	Echs1,Mdh1,Ldhb
intracellular transport	GO:0046907	3(21)	657(22938)	2,12E-02	Slc25a10,Vdac1,Tgfb3
carbohydrate metabolic process	GO:0005975	3(21)	674(22938)	2,27E-02	Dgat2,Mdh1,Ldhb
apoptosis	GO:0006915	3(21)	791(22938)	3,42E-02	Vdac1,Cdkn2c,Tgfb3

programmed cell death	GO:0012501	3(21)	799(22938)	3,51E-02	Vdac1,Cdkn2c,Tgfb3
cell death	GO:0008219	3(21)	827(22938)	3,83E-02	Vdac1,Cdkn2c,Tgfb3
ion transport	GO:0006811	3(21)	873(22938)	4,39E-02	Slc36a2,Slc25a10,Vdac1

Common down-regulated genes (in HCO and LF-HCO)

Gene Ontology category (levels 3-6)	*Items*	*S*	*TS*	*Hyp*	*Genes*
response to steroid hormone stimulus	GO:0048545	4(25)	237(22938)	1,19E-04	Insig1,Cdo1,Pparg,Ldha
response to organic substance	GO:0010033	6(25)	748(22938)	1,23E-04	Insig1,Cdo1,Atp5g3,Uqcrfs1,Pparg,Ldha
generation of precursor metabolites and energy	GO:0006091	4(25)	335(22938)	4,44E-04	Atp5o,Atp5g3,Uqcrfs1,Ldha
response to peptide hormone stimulus	GO:0043434	3(25)	188(22938)	1,09E-03	Insig1,Cdo1,Pparg
response to wounding	GO:0009611	4(25)	545(22938)	2,68E-03	Pparg,Gas6,Ddt,Cfb
response to external stimulus	GO:0009605	5(25)	913(22938)	2,70E-03	Pparg,Ldha,Gas6,Ddt,Cfb
oxidation reduction	GO:0055114	4(25)	564(22938)	3,03E-03	Cyb5r3,Cdo1,Uqcrfs1,Ldha
inflammatory response	GO:0006954	3(25)	295(22938)	3,93E-03	Pparg,Ddt,Cfb
cellular lipid metabolic process	GO:0044255	4(25)	623(22938)	4,33E-03	Dgat1,Cyb5r3,Insig1,Pparg
response to drug	GO:0042493	3(25)	306(22938)	4,35E-03	Uqcrfs1,Pparg,Ldha
lipid metabolic process	GO:0006629	4(25)	737(22938)	7,81E-03	Dgat1,Cyb5r3,Insig1,Pparg
heterocycle metabolic process	GO:0046483	3(25)	424(22938)	1,07E-02	Atp1a4,Atp5o,Atp5g3
defense response	GO:0006952	3(25)	546(22938)	2,09E-02	Pparg,Ddt,Cfb
alcohol metabolic process	GO:0006066	3(25)	546(22938)	2,09E-02	Cyb5r3,Insig1,Ldha
carboxylic acid metabolic process	GO:0019752	3(25)	567(22938)	2,31E-02	Cdo1,Pparg,Ldha
organic acid metabolic process	GO:0006082	3(25)	568(22938)	2,32E-02	Cdo1,Pparg,Ldha
regulation of programmed cell death	GO:0043067	3(25)	642(22938)	3,18E-02	Serpinb9,Hspb1,Ldha
cation transport	GO:0006812	3(25)	668(22938)	3,51E-02	Atp1a4,Atp5o,Atp5g3

Specific down-regulated genes (only in HCO)

Gene Ontology category (levels 3-6)	*Items*	*S*	*TS*	*Hyp*	*Genes*
cellular amino acid and derivative metabolic process	GO:0006519	3(30)	356(22938)	1,10E-02	Gstk1,Ivd,Fah
ion transport	GO:0006811	4(30)	873(22938)	2,60E-02	Kcnn3,Kcnh1,Atp5i,Tst

Specific down-regulated genes (only in LF-HCO)

Gene Ontology category (levels 3-6)	*Items*	*S*	*TS*	*Hyp*	*Genes*
carboxylic acid metabolic process	GO:0019752	5(20)	566(22938)	1,02E-04	Psat1,Asrgl1,Ptges,Gpd1,Ghr
organic acid metabolic process	GO:0006082	5(20)	567(22938)	1,03E-04	Psat1,Asrgl1,Ptges,Gpd1,Ghr
amine metabolic process	GO:0009308	4(20)	395(22938)	3,37E-04	Psat1,Asrgl1,Aoc3,Ghr
lipid metabolic process	GO:0006629	5(20)	735(22938)	3,46E-04	Ptges,Plin,Lpin1,Gpd1,Ghr
cellular amino acid and derivative metabolic process	GO:0006519	3(20)	357(22938)	3,50E-03	Psat1,Asrgl1,Ghr
oxidation reduction	GO:0055114	3(20)	564(22938)	1,23E-02	Etfb,Aoc3,Gpd1
cellular lipid metabolic process	GO:0044255	3(20)	622(22938)	1,60E-02	Ptges,Gpd1,Ghr
response to organic substance	GO:0010033	3(20)	748(22938)	2,60E-02	Ptges,Lpin1,Ghr
response to external stimulus	GO:0009605	3(20)	912(22938)	4,31E-02	Fgg,Ptges,Ghr

Specific down-regulated genes (only in LF-HOO)

Gene Ontology category (levels 3-6)	*Items*	*S*	*TS*	*Hyp*	*Genes*
ion transport	GO:0006811	3(9)	873(22938)	0,00388	Slc34a2,Slc25a1,Scnn1g

Specific up-regulated genes (only in LF-HOO)

Gene Ontology category (levels 3-6)	*Items*	*S*	*TS*	*Hyp*	*Genes*
regulation of signal transduction	GO:0009966	3(19)	734(22938)	2,16E-02	Sipa1,Lef1,Il6ra
positive regulation of metabolic process	GO:0009893	3(19)	748(22938)	2,27E-02	Lef1,Klf2,Il6ra
response to organic substance	GO:0010033	3(19)	748(22938)	2,27E-02	Hmgcs2,Lef1,Il6ra
regulation of cell communication	GO:0010646	3(19)	906(22938)	3,71E-02	Sipa1,Lef1,Il6ra

[a]: *enriched Gene Ontology caterogies*
[b]: *number of genes in the input list that have been matched in the enriched set and the total number of genes in the list*
[c]: *number of genes in the reference list that match with the enriched set and total number of genes in the reference list*
[d]: *pvalue calculated by the hypergeometric function*
[e]: *the genes of the input list that match with the enriched items*
